# Hypoxia, but Not Normoxia, Reduces Effects of Resveratrol on Cisplatin Treatment in A2780 Ovarian Cancer Cells: A Challenge for Resveratrol Use in Anticancer Adjuvant Cisplatin Therapy

**DOI:** 10.3390/ijms24065715

**Published:** 2023-03-16

**Authors:** Agnieszka Synowiec, Klaudia Brodaczewska, Gabriel Wcisło, Aleksandra Majewska, Agata Borkowska, Aleksandra Filipiak-Duliban, Aleksandra Gawrylak, Kinga Wilkus, Katarzyna Piwocka, Agata Kominek, Halina Waś, Sławomir Lewicki, Jacek Siewiera, Cezary Szczylik, Jolanta Szenajch, Jacek Z. Kubiak, Claudine Kieda

**Affiliations:** 1 Laboratory of Molecular Oncology and Innovative Therapies, Military Institute of Medicine-National Research Institute, 04-141 Warsaw, Poland; 2 Department of Oncology, Centre of Postrgraduate Medical Education, 01-813 Warsaw, Poland; 3 European Health Centre, 05-400 Otwock, Poland; 4 Postgraduate School of Molecular Medicine (SMM), Warsaw Medical University, 02-091 Warsaw, Poland; 5 College of Inter-Faculty Individual Studies in Mathematics and Natural Sciences, University of Warsaw, 2C Banacha Str., 02-097 Warsaw, Poland; 6 Laboratory of Cytometry, Nencki Institute of Experimental Biology, Polish Academy of Sciences, 02-093 Warsaw, Poland; 7 Institute of Outcomes Research, Maria Sklodowska-Curie Medical Academy, 00-136 Warsaw, Poland; 8 Clinical Department of Hyperbaric Medicine, Military Institute of Medicine-National Research Institute, 04-141 Warsaw, Poland; 9 Institut de Génétique et du Développement de Rennes (IGDR), CNRS, UMR 6290, Rennes University, 35043 Rennes, France; 10 Centre for Molecular Biophysics, UPR 4301 CNRS, 45071 Orleans, France

**Keywords:** ovarian cancer, cisplatin, resveratrol, normoxia, hypoxia, epithelial–mesenchymal transition markers, HIF-1α, VEGF

## Abstract

Natural compounds, such as resveratrol (Res), are currently used as adjuvants for anticancer therapies. To evaluate the effectiveness of Res for the treatment of ovarian cancer (OC), we screened the response of various OC cell lines to the combined treatment with cisplatin (CisPt) and Res. We identified A2780 cells as the most synergistically responding, thus optimal for further analysis. Because hypoxia is the hallmark of the solid tumor microenvironment, we compared the effects of Res alone and in combination with CisPt in hypoxia (pO_2_ = 1%) vs. normoxia (pO_2_ = 19%). Hypoxia caused an increase (43.2 vs. 5.0%) in apoptosis and necrosis (14.2 vs. 2.5%), reactive oxygen species production, pro-angiogenic HIF-1α (hypoxia-inducible factor-1α) and VEGF (vascular endothelial growth factor), cell migration, and downregulated the expression of ZO1 (zonula occludens-1) protein in comparison to normoxia. Res was not cytotoxic under hypoxia in contrast to normoxia. In normoxia, Res alone or CisPt+Res caused apoptosis via caspase-3 cleavage and BAX, while in hypoxia, it reduced the accumulation of A2780 cells in the G2/M phase. CisPt+Res increased levels of vimentin under normoxia and upregulated SNAI1 expression under hypoxia. Thus, various effects of Res or CisPt+Res on A2780 cells observed in normoxia are eliminated or diminished in hypoxia. These findings indicate the limitations in using Res as an adjuvant with CisPt therapy in OC.

## 1. Introduction

Ovarian cancer (OC) causes high mortality despite novel molecular diagnostics, imaging, and screenings. The main pitfalls in OC treatments come from late diagnosis due to the lack of early symptoms. According to the Global Cancer Statistics GLOBOCAN 2020, OC is the eighth most diagnosed cancer and ranks eighth in cancer mortality among women worldwide [1]. The FIGO (International Federation of Gynecology and Obstetrics) staging system indicates that 75% of such patients are diagnosed in advanced stage III or IV. Therefore, the overall 5-year survival is about 49%, while only about 30% for patients diagnosed in advanced stages [2,3]. Cytoreductive surgery and adjuvant chemotherapy based on platinum analogs (cisplatin (CisPt) or carboplatin) and taxanes (paclitaxel or docetaxel) are standard treatments [4,5,6,7]. CisPt damages the DNA in proliferating cells by crosslinking. It induces the apoptosis of cells that respond to the cell cycle checkpoints. CisPt derivatives are used for OC treatment, despite the frequent development of resistance that reduces its efficiency [8].

Natural plant compounds (phytochemicals) with anticancer properties (e.g., flavonoids, stilbenes, isoflavones), such as resveratrol (Res), curcumin, or berberine, all derived from Chinese herbal medicine, raised a great interest in anticancer therapies as adjuvants potentially increasing the efficacy of classical anticancer treatments [9,10,11]. In preclinical trials, mostly performed on cancer cell lines in standard cell culture conditions, i.e., in normoxia, these natural compounds induced apoptosis, inhibited angiogenesis, metastasis, and/or reversed multidrug resistance (reviewed by Nie et al.) [12]. Res (trans-3,5,4′-trihydroxystilbene) is a natural polyphenolic phytoalexin with a stilbene structure. Its major sources are grapes, peanuts, and blueberries [13]. Its biological activities, including its antioxidant properties, make it potentially useful not only against cancers, but also against cardiac, Alzheimer’s, Parkinson’s and metabolic diseases such as diabetes [14,15]. Widely documented in cancer and leukemia treatments [16], Res affects three stages of carcinogenesis [17], modulates signal transduction controlling cell division and growth, and as such, it should inhibit tumor cell proliferation, adhesion, invasion, metastasis and reduce inflammation [18,19,20]. Res is an antioxidant, inhibiting the production of reactive oxygen species (ROS), suppressing cyclooxygenase action, and modulating the cell cycle, apoptosis, and inflammation [18,21,22,23,24]. Furthermore, in human OC and renal carcinoma cells, Res inhibits angiogenesis via the decreased expression of HIF-1α (hypoxia-inducible factor-1α) and vascular endothelial growth factor (VEGF) [25,26], which indicates its potential against angiogenesis-mediated metastases [27,28,29]. The problem is that these effects were shown in conditions that do not reflect the lack of oxygen typical for solid tumors.

Hypoxia, the “lower-than-physiologic” level of oxygen tension (pO_2_), is a hallmark of tumors and reduces the effectiveness of chemotherapies [30,31]. Hypoxia modulates cell reactions, phenotypic changes, growth adaptation, immunosuppression, and drug resistance that occur through the stemness evolution of cells. This, in turn, lowers apoptosis and triggers the epithelial to mesenchymal transition (EMT)**,** favoring peritoneal dissemination and tumor progression [32,33]. Hypoxia-induced changes participate in apoptosis, necrosis, cell cycle arrest, differentiation, stress adaptation, and tissue remodeling [34], promoting aggressiveness and metastasis [35]. Hypoxia is an important cause of the angiogenic switch during tumor progression. It produces and sustains rapid and abnormal angiogenesis by direct induction of HIFs transcription factors for pro-angiogenic molecules, such as VEGFs [36]. Under extreme hypoxia, as upon antiangiogenic treatments, the restricted level of oxygen and HIF-1 overexpression are major factors of the selection pressure for a subpopulation of therapy-resistant cancer cells, which give rise to cancer stem cells (CSCs). CSCs repopulate the tumor and produce metastases [37]. Consequently, the cancer progression can be considered as hypoxia dependent. This prompted us to analyze the effects of hypoxia on OC treatments with CisPt and Res separately and in combination (CisPt+Res), focusing on the A2780 cell line selected for its reactive properties to these treatments in normoxia conditions.

Here, we investigated the anti-cancer, antiproliferative, and proapoptotic properties of Res alone and in combination with CisPt under normoxic vs. hypoxic conditions in the human A2780 OC line. Importantly, these cells were derived from a patient who was not drug-resistant. Therefore, it excluded potential interference of the drug resistance with our results. To our knowledge, our work is the first study of the Res effects under hypoxic conditions on CisPt-treated OC cells, which are not drug-resistant. For these reasons, our results, challenging the use of Res in adjuvant therapy with CisPt, contribute important information for further investigation into efficient treatments of OC.

## 2. Results

### 2.1. Selection of A2780 Cells for Further Study

Synergistic or antagonistic effects between Res and CisPt were assessed for five OC cell lines—A2780, TOV-21G, OAW-42, ES-2, and TOV-112D—that differ in their origin in tumor histology (carcinoembryonic vs. endometrioid, or ascitic (cystadenocarcinoma), and in the patient’s chemotherapy treatment history before cell isolation. Only the OAW-42 cell line was isolated from a patient treated with CisPt. The other cell lines were isolated from untreated patients (Table S1). Cell survival was analyzed in increasing concentrations of CisPt or Res alone. A detailed description and numerical calculations are presented in the Supplementary Methods, Figure S1, Table S2. The IC_50_ values of CisPt and Res single treatment (Table S3) indicates that the TOV-112D cell line was the most sensitive to CisPt (IC_50_ 3.25 ± 0.16 µM), while ES-2 cells were the most sensitive to Res (IC_50_ 74.32 ± 4.27 µM).

**Figure 1:**
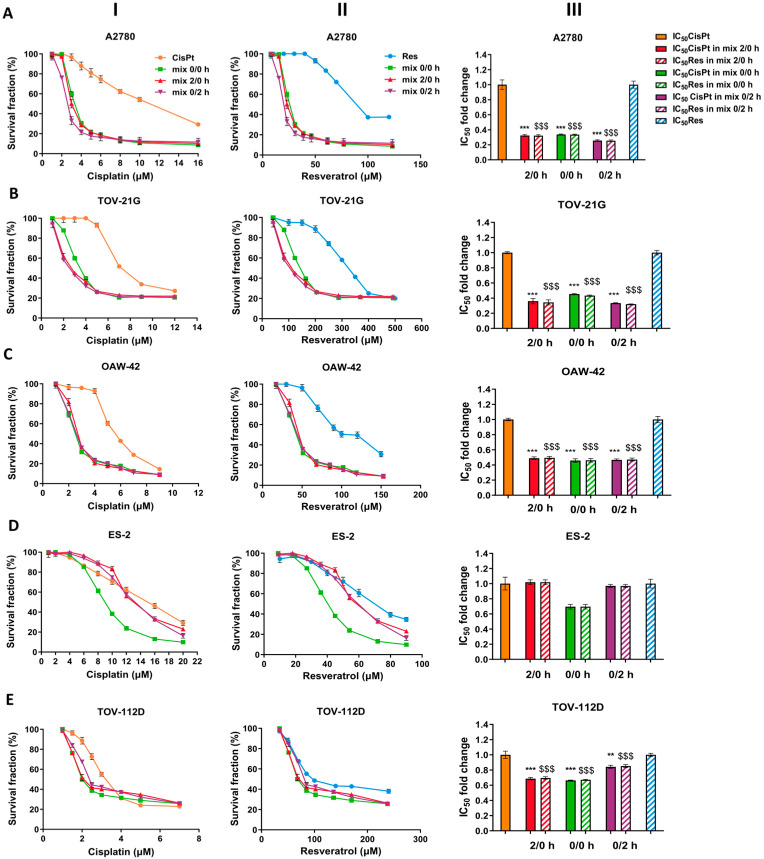
Effects of cisplatin, resveratrol, and combinations of the two drugs on OC cell line viability, estimated by alamarBlue assay. The human ovarian cancer cells were treated with cisplatin (CisPt) alone, resveratrol (Res) alone, and their combination (CisPt+Res) at a constant ratio (IC_50_′s ratio CisPt:Res) for 72 h. For combination studies, three different cell treatment protocols were used: both compounds were added at the same time (mix 0/0 h), Res given 2 h before CisPt (mix 2/0 h), or given 2 h after CisPt (mix 0/2 h). Survival curves of (**A**) A2780; (**B**) TOV-21G; (**C**) OAW-42; (**D**) ES-2; (**E**) TOV-112 cell lines; (**I**) CisPt alone vs. CisPt+Res; and (**II**) Res alone vs. CisPt+Res; (**III**) Changes of IC_50_ doses of CisPt and Res in mixes 2/0 h, 0/0 h, and 0/2 h, respectively. The results of three experiments are presented as mean ± SD. Statistically significant (Student’s *t*-test and Mann–Whitney U test) changes of IC_50_ in mixes compared to CisPt alone treatment, *** *p* < 0.001, ** *p* < 0.01; and ^$$$^ *p* < 0.001, compared to Res alone treatment.

The putative synergy between CisPt and Res treatment in normoxia was based on the values of the constant ratio of CisPt to Res (1:8.7 in A2780, 1:43 in TOV-21G, 1:17 in OAW-42; 1:5.5 in ES-2, and 1:32 in TOV-112D cell lines), determined from IC_50_ values of the single drug treatment. Next, we calculated the changes in IC_50_ values after treatment of OC cells with CisPt+Res. As shown in Figure 1 (I-III A-E) and Table S3, CisPt+Res treatment caused the reduction of IC_50_ values in comparison to IC_50_ values of CisPt and of Res alone for all protocols and OC cell lines except ES-2 cells. The highest decrease (by about 3-fold) of IC_50_ values for CisPt with Res was observed for A2780 cells.

**Figure 2:**
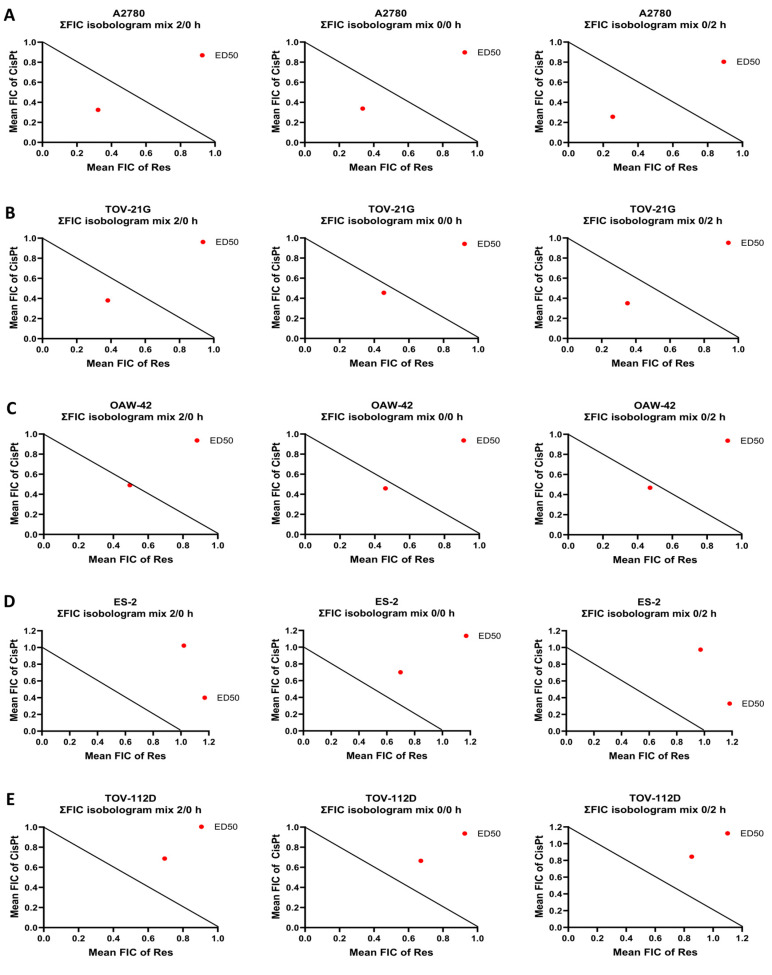
Combination effect of cisplatin with resveratrol in OC cell lines in normoxia. Isobolograms of cisplatin (CisPt) with resveratrol (Res) combination at the ED50 effect dose (median effect dose to inhibit 50% growth cells) for (**A**) A2780; (**B**) TOV-21G; (**C**) OAW-42; (**D**) ES-2; and (**E**) TOV-112 OC cells for three different cell treatment protocols: both compounds were added at the same time (mix 0/0 h), Res given 2 h before CisPt (mix 2/0 h), or given 2 h after CisPt (mix 0/2 h). The individual doses of CisPt and Res to achieve 50% growth inhibition (black straight line) were plotted on the x- and y-axes. Isobolograms show a synergistic effect between drugs when the red data point was located below the straight line, an antagonistic effect when the red data point was located above the straight line, and an additive effect when this point was located on the line. The results of three experiments are presented.

Subsequently, to assess the combinatorial efficacy of CisPt and Res, defined as synergism, additive effect, or antagonism, we generated dose–effect curves, median-effect plots, isobolograms, and combination index (CI) using the CalcuSyn software v.2.0 (Figure 2A–E and Figure S2A–E, Table S3). According to the data summarized in Table S3 and presented in the isobolograms (Figure 2A–E and Figure S2A–E lower panel), the CisPt in combination with Res exhibited the synergism (CI < 1) only in A2780 cells (CI values range, 0.67–0.51 for all protocols). We also noted a moderate synergism to the additive effect (CI = 1) in TOV-21G cells and an additive effect in the OAW-42 cells. The antagonism (CI > 1) occurred in ES-2 and TOV-112D cells. 

The above results confirmed that the strongest synergism of CisPt with Res (CisPt+Res) affects the A2780 cells in normoxia, irrespective of the drug application protocol. To minimize the excessive cytotoxic effect of CisPt and Res on cell growth, we chose to use IC_25_ doses of the drugs when used in combination (CisPt+Res). Furthermore, both compounds were added to A2780 cells at the same time (0/0 h protocol). Further, we asked whether the properties of these cells in response to CisPt, Res, or CisPt+Res treatments change under hypoxia as compared to normoxia.

### 2.2. Hypoxia-Induced Cell Death upon CisPt-Treatment and Its Modification by Res in A2780 Cells

**Figure 3:**
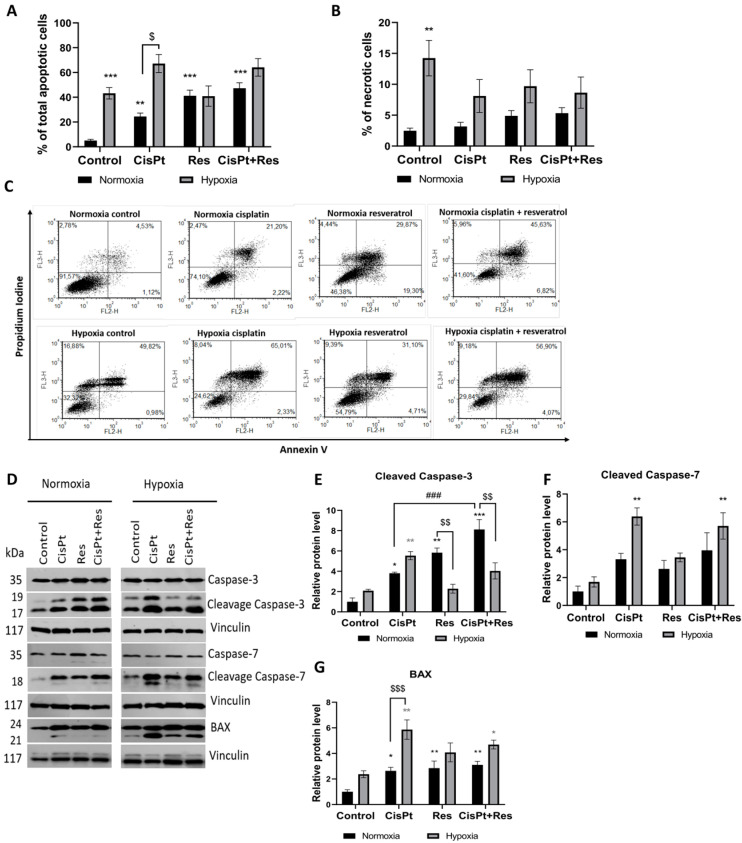
Analysis of cisplatin, resveratrol, and in combination to induce apoptosis in OC cells under normoxic and hypoxic conditions by flow cytometry and Western blot. A2780 cells were treated with cisplatin (CisPt) (5.9 µM), resveratrol (Res) (63 µM), and in the combination CisPt+Res (at the same concentrations) according to Section 4.4: Establishment of Hypoxic Culture Conditions and Experimental Outline. (**A**) Percentage of apoptotic cells; (**B**) percentage of necrotic cells; (**C**) representative histograms from apoptosis analysis in A2780 cells; (**D**–**G**) Western blot analysis of cleaved caspase-3, cleaved caspase-7, and BAX protein expression. The graphs represent the densitometric analysis of the immunoblots. Vinculin was used as a loading control and normalized to the normoxia control as 1. The results shown are the mean ± SEM of three independent experiments, with *** *p* < 0.001, ** *p* < 0.01, * *p* < 0.05, vs. normoxia control (black asterisk) and vs. hypoxia control (grey asterisk); ^$$$^ *p* < 0.001, ^$$^ *p* < 0.01, ^$^ *p* < 0.05 comparing the normoxia group with the hypoxia group; and ^###^ *p* < 0.001, CisPt alone vs. CisPt+Res.

To determine the effect of hypoxia on cell survival (apoptosis and necrosis), we treated A2780 cells with CisPt, Res, or CisPt+Res under normoxic and hypoxic conditions. In normoxia, treatment with CisPt (*p* = 0.005), Res (*p* < 0.0001), and CisPt+Res (*p* < 0.0001) significantly increased apoptosis (Figure 3A,C). Thus, we assessed, by Western blot, the level of expression of selected apoptosis-related proteins. As shown in Figure 3D,E,G, under normoxia, the CisPt significantly upregulated the expression of cleaved caspase-3 (*p* = 0.034) and BAX (*p* = 0.02) proteins. Similarly, Res alone significantly increased the levels of cleaved caspase-3 (*p* = 0.0015) and BAX (*p* = 0.0085). Res also increased the CisPt-induced cell death (*p* = 0.0014) and the expression of cleaved caspase-3 (*p* = 0.003). CisPt+Res significantly increased the level of the caspase-3 (*p* = 0.036), while the CisPt alone decreased the level of caspase-7 (*p* = 0.029) (Figure 3D and Figure S3A,B). We also analyzed the percentage of necrotic A2780 cells and observed a slight increase in necrosis in cells treated with CisPt, Res, or CisPt+Res in normoxia (Figure 3B).

To mimic the natural tumor microenvironment in our in vitro conditions, we exposed A2780 cells to a low pO_2_ (1%). The pO_2_ level in the normoxic or hypoxic culture medium was measured with an ABL90 FLEX analyzer, showing that the pO_2_ values under normoxic and the hypoxic conditions were stable for 6 days (Table 1). Under the hypoxic condition, the medium had a 1% pO_2_, i.e., equivalent to ~15 mmHg, which is clinically relevant to tumor hypoxia [38]. It was shown that, in follicular fluid, the oxygen level can vary from 11 to 80 mmHg [39,40]. Upon prolonged (5 days) hypoxia, the A2780 cell viability dramatically decreased by apoptosis (*p* = 0.0006), without significantly increasing the levels of both cleaved caspase-3,-7, and the pro-apoptotic protein BAX (Figure 3A,C–H). Apoptosis increased after the treatment with CisPt (*p* = 0.1), in parallel with the induction of cleaved caspase-3 (*p* = 0.0053), cleaved caspase-7 (*p* = 0.0029), and BAX (*p* = 0.0028) (Figure 3A,C–G). The A2780 cells did not respond to Res, but the CisPt+Res treatment increased the level of BAX (*p* = 0.05) and cleaved caspase-7 (*p* = 0.007) in hypoxia. Additionally, hypoxia itself significantly increased the percentage of necrotic A2780 cells (*p* = 0.0041) (Figure 3B).

Altogether, hypoxia significantly induced apoptosis and necrosis in A2780 cells. Surprisingly, Res caused an increase in the percentage of apoptotic cells in normoxia, but not in hypoxia. This was the first indication that hypoxic vs. normoxic conditions can modify the effects of Res. Similarly, normoxia significantly induced apoptosis via cleaved caspase-3 and BAX. In contrast, the treatment of A2780 cells with Res under hypoxic conditions did not affect the level of cleaved caspase-3 protein and slightly increased BAX protein expression, which suggests that mechanisms other than apoptosis are involved in the observed cell death. 

### 2.3. Res Reduces Accumulation of A2780 Cells in the G2/M Phase under Hypoxia but Does Not Impact CisPt-Induced Inhibition of Cell Proliferation

**Figure 4:**
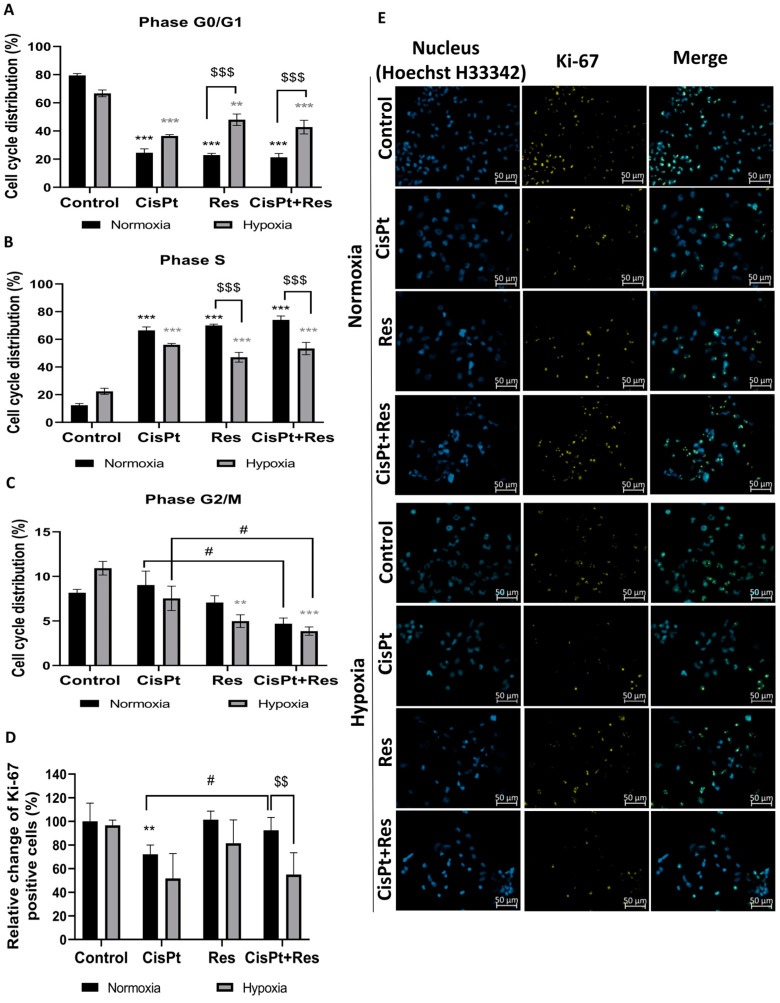
Modification in the distribution of the cell cycle phases and Ki-67 expression after treatment of A2780 cells with cisplatin, resveratrol, and in combination in normoxia and hypoxia. The cells were treated according to Section 4.4: Establishment of Hypoxic Culture Conditions and Experimental Outline, before staining with propidium iodide (PI). The percentage of cells in a given phase of the cell cycle was analyzed by flow cytometry. Graphs show the percentage of cells in the (**A**) phase G0/G1; (**B**) the S phase; (**C**) the G2/M phase of the cell cycle. Panel (**D**) shows the relative change of Ki-67 positive cell percentage compared to the 100% normoxia control. (**E**) Representative images of Hoechst nuclear staining and Ki-67 staining after treatment with cisplatin (CisPt), resveratrol (Res), and CisPt+Res (Zeiss Axio Observer, magnification 40×, scale bar is equal to 50 µm). Data are representative of at least three repeats. The results are presented as the mean ± SEM values. *** *p* < 0.001, ** *p* < 0.01, vs. normoxia control (black asterisk) and grey asterisk vs. hypoxia control; ^$$$^ *p* < 0.001, ^$$^ *p* < 0.001, comparing the normoxia group with hypoxia group; and ^#^ *p* < 0.05, CisPt alone vs. CisPt+Res.

Because cell cycle regulation in cancer cell treatment is crucial for apoptosis induction, we measured the effects of drug treatment on the cell cycle phase distribution of A2780 cells in normoxia and hypoxia using flow cytometry. As shown in Figure 4A–C, CisPt and Res alone resulted in the accumulation of these cells in the S phase of the cell cycle (*p* < 0.001) with the concomitant decrease of the number the cells in the G0/G1 phase (*p* < 0.001) in normoxia. However, there was no additive effect of Res and CisPt (CisPt+Res). Hypoxia itself did not affect the cell cycle course, although Res alone or in combination with CisPt significantly decreased the G2/M phase proportion (*p* = 0.0012) and (*p* = 0.0002), respectively, as compared to the control and to CisPt-treated cells (*p* = 0.04).

To check the effects of hypoxia on CisPt, Res, and CisPt+Res treatments’ alteration of cell proliferation, we measured the expression of proliferation marker Ki-67. Figure 4D,E shows that cytotoxicity was concomitant with the decrease of Ki-67 level in A2780 cells exposed to CisPt under normoxia (*p* = 0.0017) and hypoxia (*p* = 0.0503). Res alone did not affect the Ki-67 level in both oxygen conditions, but reversed CisPt-downregulated proliferation (*p* = 0.016) only in normoxia. The CisPt-induced decrease in the Ki-67 level persisted after the addition of Res (*p* = 0.06) under hypoxic conditions.

In summary, Res differentially affects A2780 cells’ sensitivity dependent on oxygen availability. The synergistic effect of CisPt+Res occurred only in normoxia, demonstrating how oxygen availability changes cancer cell responses to the treatments, and that, in hypoxia, characteristic for solid tumors, treatment with Res does not reduce OC cell proliferation rate.

### 2.4. Hypoxia Induces ROS Generation and Res Represses This Effect during Short-Time Exposure of A2780 Cells

**Figure 5:**
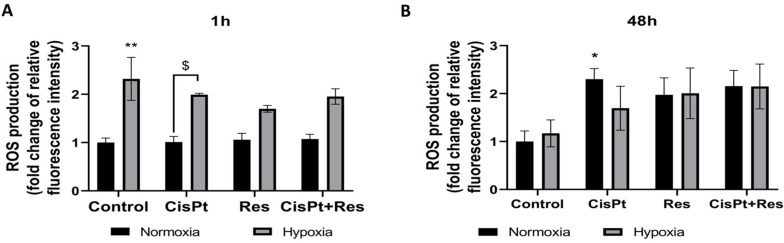
Hypoxia-dependent production of intracellular reactive oxygen species in A2780 cells upon treatment with cisplatin, resveratrol, and in combination. The cells were treated with cisplatin (CisPt), resveratrol (Res), and both in combination (CisPt+Res) according to Section 4.4: Establishment of Hypoxic Culture Conditions and Experimental Outline. The intracellular ROS were assayed by 5 μM DCF-DA staining for 30 min. at 37 °C, followed by flow cytometry analysis. (**A**) ROS production after one hour; (**B**) after 48 h treatment with drugs. The results of three independent experiments are presented as mean values ± SEM, normalized to the normoxia control as 1. ** *p* < 0.01, * *p* < 0.05 vs. normoxia control; ^$^ *p* < 0.05, CisPt in normoxia vs. CisPt in hypoxia.

To test whether A2780 cells sensitivity to Res under hypoxia was related to cytoprotective and antioxidant activity, we measured the production of intracellular reactive oxygen species (ROS) using a DCFDA assay. This assay detects mostly cellular hydrogen peroxide, and to a lesser extent hydroxyl radicals, hydroperoxides, or peroxynitrites [41]. The A2780 cells were treated with CisPt, Res, and Cispt+Res for one hour and for 48 h under normoxic and hypoxic conditions. A short (1 h) exposure of control cells to low pO_2_ significantly increased (*p* = 0.0025) production of ROS. Treatments of A2780 cells with the studied drugs did not change the ROS production under normoxic conditions (Figure 5A).

As shown in Figure 5B, the long (48 h) exposure of control A2780 cells to hypoxia did not induce significant changes in the ROS level. The cytotoxic effect of CisPt under normoxic conditions was accompanied by a significant increase in ROS production (*p* = 0.05), and a similar tendency was observed in the presence of Res (0.15) and CisPt+Res (*p* = 0.08). Hypoxia increased the level of ROS, regardless of which drug was applied.

Hypoxia-induced ROS production in control A2780 cells was clearly visible after a short-time exposure, while treatments with CisPt, Res, and CisPt+Res for 48 h induced the increase of ROS production in both oxygen conditions. Therefore, cytotoxic effects seem to be related to ROS-induced stress and not modulated by the potential antioxidant activity of Res.

### 2.5. Hypoxia Induces a Proangiogenic Response in A2780 Cells

**Figure 6:**
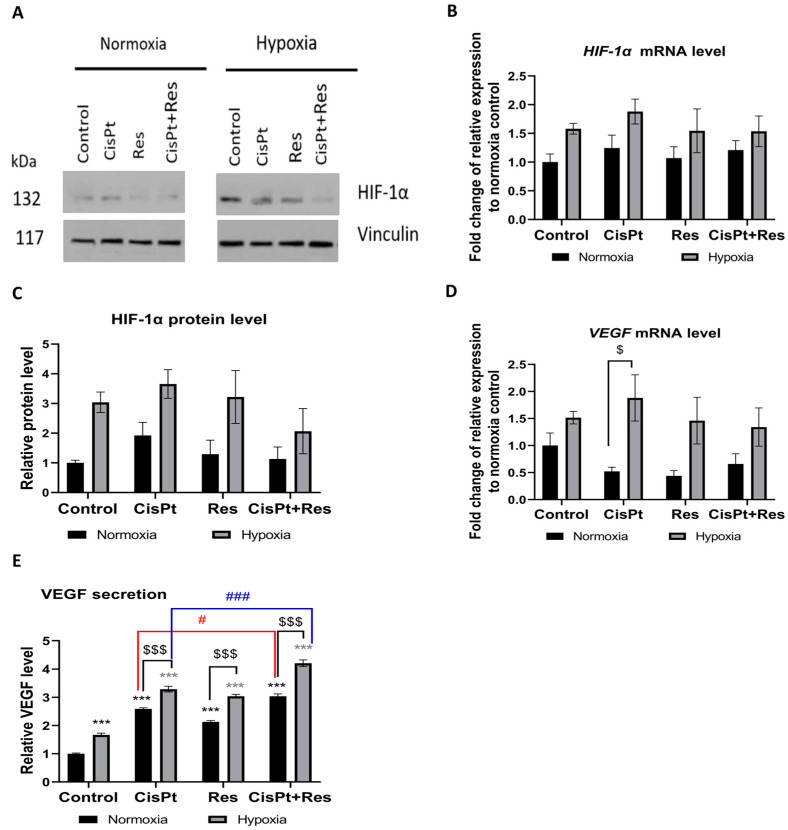
Hypoxia-modified *HIF-1α* and *VEGF* mRNA and protein expression upon cisplatin, resveratrol, and the combination treatments. A2780 cells were treated with cisplatin (CisPt), resveratrol (Res), and the combination (CisPt+Res) according to Section 4.4: Establishment of Hypoxic Culture Conditions and Experimental Outline. (**A**) Immunoblots of HIF-1α protein; (**B**) the level of *HIF-1α* mRNA expression was measured by RT-qPCR and normalized to PPIA and GUSB expressions and normoxia control as 1; (**C**) densitometric analysis of HIF-1α protein. Vinculin was used as a loading control and normalized to the normoxia control as 1; (**D**) the level of *VEGF* mRNA expression was measured by RT-qPCR and normalized to PPIA and GUSB expressions and the normoxia control as 1; (**E**) secretion of VEGF by A2780 cells was measured by the ELISA method and normalized to the normoxia control as 1. The results shown are the mean ±SEM of three independent experiments, with *** *p* < 0.001, vs. normoxia control (black asterisk) and vs. hypoxia control (grey asterisk); ^$$$^ *p* < 0.001, ^$^ *p* < 0.05, comparing the normoxia group with hypoxia group; and ^###^ *p* < 0.001, ^#^ *p* < 0.05 CisPt alone vs. CisPt+Res.

Because hypoxia promotes angiogenesis, we assessed the levels of the expression of the HIF-1α gene and its downstream target gene VEGF. As shown in Figure 6A–C, and as expected, the exposure of control A2780 cells to hypoxia for 5 days induced, though not statistically significant, the expression of HIF-1α mRNA and protein.

As the VEGF gene plays a major role in tumor angiogenesis in response to hypoxia, we assessed whether the studied drugs could inhibit VEGF mRNA expression and protein secretion. Our analysis confirmed that hypoxia insignificantly increased the *VEGF* mRNA level in A2780 cells as compared to normoxia (Figure 6D). However, as shown in Figure 6E, the production of pro-angiogenic VEGF was upregulated by the tested drugs in both oxygen conditions. We noted that hypoxia itself upregulated the VEGF secretion (*p* = 0.0002), and this significantly increased upon CisPt, Res, and CisPt+Res treatments in normoxia and hypoxia. Moreover, the additive effect of Res on CisPt-induced VEGF secretion was observed both in normoxic (*p* = 0.0016) and hypoxic (*p* = 0.0004) conditions (Figure 6E).

### 2.6. CisPt, Res, and CisPt+Res Differently Affect EMT Markers under Hypoxic and Normoxic Conditions

To detect potential manifestations of EMT in A2780 cells, we analysed the effects of hypoxia and CisPt, Res, or CisPt+Res treatments on the morphology of A2780 cells. As shown in Figure S4, in normoxia, the untreated cells form tight clusters, and individual cells show epithelial cobblestone-like morphology. Treatment with CisPt caused cell elongation and spindle-like or fibroblast-like morphology similar to that observed by Baribeau et al. [42]. This phenotype was less prominent after treatment with Res alone or CisPt+Res. However, hypoxia induced morphological changes in (a) pre-incubated cells under hypoxic conditions (72 h), and (b) after treatment with CisPt, Res, and CisPt+Res (48 h). A2780 cells displayed more elongated, spindle-like, and fibroblast-like shapes, independently of the drug used (Figure S4). This change can be attributed to the EMT, which is well known to be induced by hypoxia in cancer cells.

**Figure 7:**
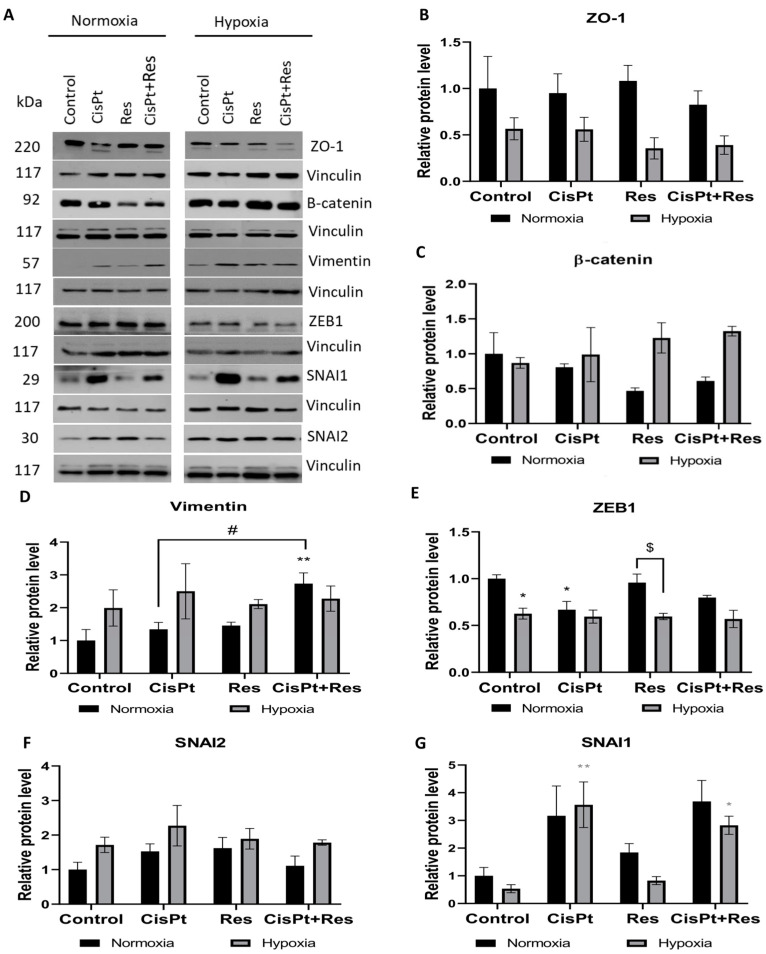
Hypoxia-dependent modifications on EMT markers in A2780 cells upon cisplatin, resveratrol, and in the combination treatments. The cells were treated with cisplatin (CisPt), resveratrol (Res), and the combination (CisPt+Res), according to Section 4.4: Establishment of Hypoxic Culture Conditions and Experimental Outline. (**A**) Immunoblots of EMT markers; (**B**–**G**) densitometric analysis of ZO-1, β-catenin, vimentin, ZEB1, SNAI2 (SLUG), and SNAI1 proteins. Vinculin was used as a loading control and normalized to the normoxia control as 1. Data are expressed as mean values ± SEM of three independent experiments with ** *p* < 0.01, * *p* < 0.05, vs. normoxia control (black asterisk) and hypoxia control (grey asterisk); ^$^ *p* < 0.05, comparing the normoxia group with hypoxia group; and ^#^ *p* < 0.05, CisPt alone vs. CisPt+Res.

To further verify the potential EMT induction, treated A2780 cells were tested for the expression of EMT markers: ZO-1 (an epithelial marker), vimentin, β-catenin, SNAI1, and SNAI2 (SLUG) proteins under our experimental conditions. Figure 7A,B shows that ZO-1 decreases in hypoxic conditions, irrespective of the drug treatment. However, none of the tested drugs affected ZO-1 expression in normoxia. Figure 7A,C demonstrates that one mesenchymal marker, the β-catenin level, was not affected by hypoxia alone, while the normoxia together with Res treatment (*p* = 0.15) decreased the expression. Hypoxia itself non-significantly increased the expression of the mesenchymal marker vimentin, irrespective of applied drugs. Vimentin level increased upon CisPt+Res treatment (*p* = 0.006), as compared to CisPt alone treatment (*p* = 0.022), while in normoxia, the Res alone treatment did not have an effect (Figure 7A,D).

We also assessed the effects of drugs and oxygen conditions on the expression of chosen transcription factors ZEB1, SNAI2, and SNAI1, which induce EMT. The expression of ZEB1 was reduced by low pO_2_ both in control (*p* = 0.02) and in drug-treated cells. In normoxia, CisPt downregulated the ZEB1 level (*p* = 0.03), but this effect was attenuated by Res in CisPt+Res treatment (Figure 7A,E). As shown in Figure 7A,F,G, the SNAI2 expression slightly increased in hypoxia, especially after CisPt treatment. Despite the known functional link between Slug and Snail, the expression of SNAI1 was differently modulated than the expression of SNAI2 (Figure 7A,G). CisPt alone (*p* = 0.19) and in combination with Res (*p* = 0.09) increased the expression levels of SNAI1 in normoxia, and this effect was enhanced in hypoxia (*p* = 0.006 and *p* = 0.03, respectively). Res alone slightly upregulated the level of SNAI1 protein in normoxia. Taken together, the decrease in ZO-1 expression and the increase in vimentin and SNAI2 expression in A2780 cells under hypoxic conditions suggests a hypoxia-dependent EMT that persisted during drug treatments.

### 2.7. Hypoxia Increases Cell Motility While Res Weakens This Effect upon Short-Time Exposure

**Figure 8:**
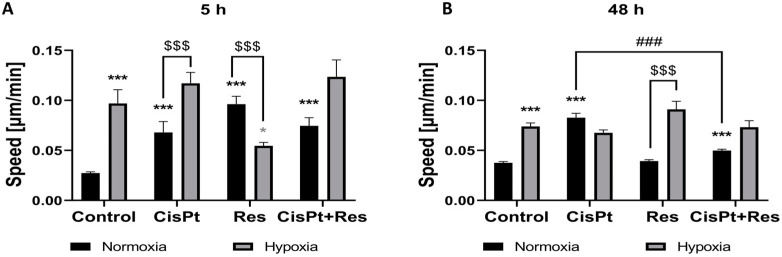
Hypoxia-dependent modification of migration of A2780 cells upon cisplatin, resveratrol, and the combination treatments. The cells were treated with cisplatin (CisPt), resveratrol (Res), and both in combination (CisPt+Res) according to Section 4.4: Establishment of Hypoxic Culture Conditions and Experimental Outline. The cell migration activity was recorded for 48 h with a time step of 20 min at 5× magnification, using Zeiss Axio Observer microscope Z1/7–software Zen 2.6 (Blue edition). The speed of 50 single non-proliferating cells after treatment with CisPt, Res, and CisPt+Res under normoxic and hypoxic conditions was analyzed (**A**) at the 5 h time-point and (**B**) at the 48 h time-point. The results of three independent replicates are presented with the mean values ± SEM (total n = 150) with *** *p* < 0.001, * *p* < 0.05, vs. normoxia control (black asterisk) and vs. hypoxia control (grey asterisk); ^$$$^ *p* < 0.001, comparing the normoxia group with the hypoxia group; and ^###^ *p* < 0.001, CisPt alone vs. CisPt+Res.

EMT is often linked to increased cell mobility. Thus, the migration rate of A2780 cells was assessed at 5 h and 48 h time-points in all treatments. In normoxia, CisPt treatment significantly increased cell migration at 5 h (*p* < 0.0001) and 48 h (*p* < 0.0001) time-points (Figure 8A,B). Res alone increased cell migration during the first 5 h (*p* < 0.0001), but it returned to the control level after 48 h of culture. This same effect could be partly observed in CisPt+Res treatment: the cell migration was statistically faster than that of control cells at both time points, but slower than after exposure to CisPt alone (*p* < 0.0001) at the 48 h time-point.

The limiting effect of Res on CisPt-induced cell migration was attenuated in hypoxia, which itself induced higher cell motility at both time points. CisPt treatment did not affect A2780 cells mobility under hypoxic conditions. The inhibitory effect of Res alone was confirmed in hypoxia only at the 5 h time-point (*p* = 0.01), and Figure 8B demonstrates that, at 48 h, the hypoxia-induced increase in the movement rate is no longer inhibited by Res. Thus, higher cell velocity observed in response to drugs and hypoxia at 48 h might be highly significant in terms of cancer cell metastatic potential and cancer aggressiveness. The incubation of cells with CisPt+Res did not seem to impact cell motility rate as compared to CisPt-treatment.

## 3. Discussion

Our study is the first demonstration of the effects of Res used in combination with CisPt in OC cells under hypoxic conditions. We showed that the biological properties of Res on A2780 ovarian cancer cells, also in combination with CisPt, are strongly dependent upon the level of oxygen tension. This demonstrates that oxygen availability influences the redox balance to which the antioxidant Res is submitted, resulting not only in the lower chemo toxic effects of Res, but also counteracting the effects of the drugs. Thus, our data challenge the usefulness of Res as an adjuvant for CisPt therapy of OC.

Several research groups tested the effects of Res and its metabolites on xenografts of tumors in nude and SCID mice [43,44,45]. However, xenografts present important limitations due to the lack of immune response in athymic nude and SCID mice, which may diverge the results from those expected for tumors in their natural environment. Additionally, Res presents a relatively low bioavailability and activity, it is rapidly metabolized, poorly water soluble, instable, and very weakly distributed in tissues, hampering its effects [46,47]. On the whole, this limits its effectiveness in humans in clinical trials [48,49]. The Res activity in animals cannot be extrapolated to humans [50] due to genetic variability, differences in anatomy, physiology, and metabolism, as well as insufficient knowledge on (a) the differences in tumor microenvironment; (b) bioeffective, nontoxic doses; (c) effective concentrations of Res in organs or tissues; (d) time of exposure to biologically active concentrations, and (e) the types of cancer sensitive to Res, which hinders the interpretation for the use of Res in clinical trials [51]. For these reasons, we believe that our in vitro cell culture analyses carried out under hypoxic vs. normoxic conditions present in the current article may better reflect the real behavior of cancer cells within a human tumor in situ, compared to studies in nude mice.

Our study on OC cell lines was focused on the potential enhancement of the anticancer drug CisPt by Res; however, it showed that the hypoxic conditions modify expected anticancer effects of Res. We reasoned that Res could potentially trigger a synergistic effect with CisPt on A2780 cells, as suggested by our screening conducted in normoxic conditions, because Res is a candidate molecule for increasing the efficacy of chemotherapies through apoptosis. Nessa et al. [52] showed that the combination of Res with chemotherapeutics might sensitize OC cells to platinum-based chemotherapy. However, this study was conducted in normoxia, while hypoxia is the principal hallmark of any solid tumor. Moreover, hypoxia rules its progression, but also its response to anticancer treatments. Thus, it has to be taken into account when assessing the ability of potential adjuvants in anticancer therapies. In addition, cytostatic drugs (e.g., CisPt or doxorubicin) may induce, in the hypoxic context, selection of resistant cells [53,54]. Therefore, the controlled hypoxic atmosphere was used in the current study to mimic the tumor microenvironment, and to obtain biologically and clinically more relevant conditions to evaluate the potential adjuvant effect of Res on the CisPt treatment of OC A2780 cells, as compared to the normoxic conditions analyzed so far.

The important property of Res is that it induces apoptosis. Here, we confirmed that Res alone induced apoptosis and significantly sensitized A2780 cells to CisPt; however, only under normoxic conditions, which is consistent with observations by Baribeau et al. [42]. This allows us to ask whether such an effect of Res is only restricted to these particular cells, or whether it applies also to other OC cells, and most importantly, to OC tumors in situ. The study by Kim et al. [55] on OC cells showed that Res increased the cleaved caspase-3 level in normoxia. Similarly, Res treatment increases cleaved caspase-3 and BAX proteins in gastric, pancreatic, prostate cancer cells, lymphoma, leukemia, and myeloma cells. However, again, all these findings were limited to normoxia [16,56]. We confirmed that CisPt and Res caused apoptosis via the caspase-3 cleavage pathway in normoxia; however, hypoxia weakened the pro-apoptotic action of Res. Cleaved caspase-7 and BAX expression follow a similar pattern: increased by CisPt and decreased by Res in low pO_2_. Therefore, hypoxic A2780 cells remained responsive to CisPt treatment, but not to Res. This suggests that distinct mechanisms trigger apoptosis in normoxia vs. hypoxia, or that mechanisms other than apoptosis are inducing cell death, e.g., autophagy, in these OC cells [57,58].

Res was also reported to induce the accumulation of cells in the G0/G1 or S phase and reduced the number of cells in the G2/M phase [59,60,61]. Our work demonstrates that, in hypoxia, Res alone and in combination with CisPt induces accumulation of A2780 cells in the S phase and decreases the number of cells in the G2/M phase. Res was shown to inhibit the proliferation of OC cells under normoxic conditions [58,62,63]. In contrast, we showed here that Res alone did not affect A2780 cell proliferation either in normoxia or hypoxia. CisPt anti-proliferative action was observed in both oxygen conditions and was reversed by treatment with Res, but again, this was exclusively in normoxia.

Cancer cells are characterized by increased ROS production, a molecular mechanism induced by hypoxia and causing oxidative stress, which induces apoptosis and tissue damage [64,65,66]. In our study, the acute low pO_2_ short-time exposure induced a high level of ROS. Therefore, we assumed that, in hypoxia, Res antioxidative effects prevailed over cytotoxicity. However, our results evidenced that the short-term exposure (1 h) to Res could not scavenge hypoxia-induced ROS. In contrast, after the long-time (48 h) exposure, both in normoxia and in hypoxia, Res increased the oxidative stress effects (as in Ref. [67]) similarly to the CisPt treatment [68]. This is consistent with results of Kim et al. [55] and Lang et al. [69], showing Res-induced ROS production in OC cells being related to ROS-dependent Notch1/PTEN/Akt signaling. Most significantly, our study demonstrates that hypoxia protects A2780 cells from Res-induced apoptosis, while applied either alone or in combination with CisPt.

One of the main regulators of the tumor microenvironment in response to hypoxia is tumor angiogenesis, the main way to bring oxygen to tissues. It is a hallmark of tumors, which turns on HIF-1α expression [70]. The overexpression of HIF-1α is associated with the shorter survival of patients with OC [71,72]. Nunes et al. showed that OC cells express higher levels of HIF-1α in hypoxia than in normoxia [73]. Our results confirm this observation in A2780 cells, regardless of the treatment applied. The angiogenic switch turning on vascularization in solid tumors is due to HIF-1α stabilization allowing the transcription of proangiogenic factors, such as VEGFs, among which a secreted VEGF-A is the main inducer of angiogenesis [74]. Overexpression of VEGF creates a permanent production of pathologic vessels, responsible for the dissemination of cancer cells, thus early recurrence of OC [75]. We confirm here that hypoxia upregulated *VEGF* mRNA expression and protein secretion independently of the drug treatment used in our analysis. Importantly, the combination CisPt+Res treatment in both oxygen conditions effected VEGF production to an even higher extent than CisPt treatment alone. This points to another important limitation in the expected positive adjuvant effects of Res.

EMT plays important roles in the cancer development, progression, generation of chemoresistance, and metastasis [76,77]. It was shown that paclitaxel-resistant OC cells display the EMT phenotype [78]. Here, we demonstrated that hypoxia decreased the expression of the EMT marker Snail, while CisPt and CisPt+Res treatment increased its level in both oxygen conditions. CisPt-increased Snail expression was reported [79,80] and was associated with the increased expression of *Slug*, leading to CisPt resistance of OC [81]. In our study, Slug protein increased under hypoxia, especially after CisPt-treatment consistent with Kurrey et al. [82]. Snail and Slug are associated with modified cell morphology and shape [83]. As observed by us, hypoxia triggers the elongation of A2780 cells and induced their spindle-like and fibroblast-like shape that paralleled the upregulation of Slug protein expression. CisPt-treatment induced the increase of SNAIL1 and SNAIL2 expression and the reshaping of A2780 cells. This suggests the EMT of these cells. Moreover, we showed here that the level of the ZEB1 transcription factor decreased upon hypoxia, regardless of the drug used. In normoxic conditions, ZEB1 significantly decreased after CisPt treatment but, importantly, this effect was not modulated by Res. Other works reported the increase of ZEB1 expression in A2780 cells upon CisPt treatment, and, similarly, no effect after adding Res [42]. Differences in CisPt-related results in our studies are likely the result of different dosage of Res and different time pints time points examined. Cai et al. showed that the increased ZEB1 expression in tissues from patients with OC correlated with the advanced stages of the disease, tumor metastasis, and lower survival rates [84]. The ZEB-1 expression correlates with the level of vimentin expression [85]. Here, we showed that, in A2780 cells, the vimentin expression is increased in hypoxia, while in normoxia, it was significantly increased only by CisPt+Res treatment.

The ZO-1 expression decrease and the increase in vimentin expression observed in our study may suggest a hypoxia-dependent EMT in A2780 cells. In contrast, increased Slug expression may induce spindle-like morphology by the loss of the apical–basal polarity in these cells [86].

ZEB1 regulates invasion and migration of breast cancer and OC cells [84,87]. Similarly to Greville et al. [88], we showed here that hypoxia itself increases the motility of A2780 cells, which may indicate the increase of their pro-metastatic potential. We initially hypothesized that Res could decrease the metastatic potential of A2780 cells via reducing the rate of their migration. However, our results indicate that this was only a short-term effect (5 h of treatment), and it disappeared during longer treatment (48 h). Oppositely, in normoxia, Res alone increased the A2780 cell motility upon a short-time exposure, but reduced migration rate in combination with CisPt after 48 h treatment as compared to CisPt-treatment. These results suggest that Res alone, but not in combination with CisPt, may show only a short-term potential in the reduction of migratory properties under hypoxic conditions.

Our results show that both hypoxia- and drug-driven cell selection may promote the survival of aggressive and metastatic cells, which might be highly significant for future treatments and assessment of treatment efficacy in patients with ovarian cancer.

## 4. Materials and Methods

### 4.1. Chemicals

Cisplatin (*cis*-Diamineplatinum(II) dichloride) and resveratrol (3,4′,5-Trihydroxy-*trans*-stilbene) were from Sigma-Aldrich (St. Louis, MO, USA). The 100 mM stock solution of Res was prepared in DMSO, and the 7.5 mM stock solution of CisPt was prepared in PBS. They were aliquoted and stored at −20 °C, and the final dilutions of the two drugs in the culture medium were prepared immediately before use.

### 4.2. Cell Culture, Drug Treatment, and Viability Assessment

The human OC cell lines TOV-21G and TOV-112D from the American Type Culture Collection (ATCC, Manassas, VA, USA) were cultured in a 1:1 mixture of MCDB 105 medium and Medium 199 (Sigma-Aldrich), supplemented with 15% (*v*/*v*) FBS (Gibco, Life Technologies, Paisley, UK). The ES-2 cells (ATTC), OAW-42 cells (Center for Translational Research and Molecular Biology of Cancer Maria Sklodowska-Curie Memorial Cancer Center and Institute of Oncology, Gliwice Branch, Poland), and A2780 cells (European Collection of Authenticated Cell Cultures, ECACC, Salisbury, UK) were cultured in RPMI 1640 medium (Gibco) containing 10% (*v*/*v*) FBS. Cells were grown at 37 °C in a humidified atmosphere of 5% CO_2_.

Cells were treated for 72 h with serial dilutions of CisPt or Res in the culture medium (200 µL per well) in triplicate. Then, the medium containing drugs was removed, and cells were cultured with a drug-free medium for 72 h to distinguish viable and proliferating cells from non-proliferating cells. Afterward, alamarBlue (Thermo Fisher Scientific, Waltham, MA, USA) was added at a final concentration of 10% (*v*/*v*) for 24 h, and absorbance was measured at 570/600 nm using a microplate reader (Bio-Tek Instruments, Winooski, VT, USA). The cell viability as the percent difference in reduction of resazurin-based solution between control and drug-treated cells was calculated according to the manufacturer’s protocol. Control cells cultured in a drug-free medium with an addition of 0.1% DMSO were used as the positive control, while the same except for a cell-free medium was the negative control. All cell lines were tested for the absence of Mycoplasma (PromoKine, Heidelberg, Germany), and the passage number did not exceed 10. Dose-dependent response curves to calculate IC_50_ values of CisPt or Res were plotted using GraphPad Prism Software v.9.0 (GraphPad Software, San Diego, CA, USA).

### 4.3. Determination of Drug Synergy in Different OC Cell Lines

The five OC cell lines were treated with serial dilutions of CisPt and Res combinations at a constant ratio (based on obtained IC_50_ values of CisPt and Res single for each cell line) according to the protocol described above. The constant combination ratio CisP:Res was 1:8.7; 1:43; 1:17; 1:5.5, and 1:32 for A2780, TOV-21G, OAW-42, ES-2, and TOV-112D cell lines, respectively.

The synergy between CisPt and Res was assessed using three different treatment approaches: (A) both compounds were added at the same time (mix 0/0 h); (B) Res was given 2 h before CisPt (mix 2/0 h); and (C) Res was given 2 h after CisPt (mix 0/2 h). To determine the combined activity of CisPt and Res, we used dose-effect analysis, isobologram plots, and the combination index (CI) method derived from the median-effect principle of Chou and Talalay [89], which was generated using the CalcuSyn software (version 2.0, Biosoft, Cambridge, UK). According to the Chou–Talalay method, CI values > 1 determine the antagonistic effect, CI = 1 indicates additive effect, and CI < 1 determines the synergy between tested drugs.

### 4.4. Establishment of Hypoxic Culture Conditions and Experimental Outline

**Figure 9:**
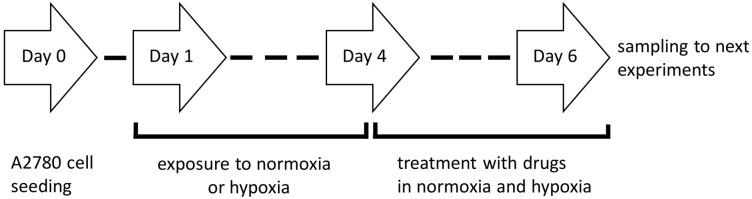
The protocol scheme for cell adaptation to experimental conditions.

To estimate the role of hypoxia, A2780 cells were seeded and initially cultured for 24 h in normoxia (in a standard incubator CO_2_, 37 °C, pO_2_~19%, 95% humidity). Then, cells were transferred to a hypoxia chamber (5% CO_2_, 37 °C, pO_2_ = 1%, 95% humidity; BioSpherix, Xvivo System Model X3, USA) or kept in normoxic conditions, and the culture medium (preconditioned under normoxia or hypoxia for 24 h before culturing) was added. After 72 h exposure of cells to normoxia or hypoxia, half of the medium was removed and replaced by fresh medium (preconditioned to the appropriate oxygen tension 72 h before adding to the culture) with CisPt, Res, or their combination (CisPt+Res) for 48 h; the general scheme of experiments is shown on Figure 9. To avoid excessive cytotoxicity, especially in drug mixtures, the compounds were used at doses of IC_25_. Cells in 0.1% DMSO medium served as a control. Hypoxic conditions (pO_2_ = 1%) were obtained in a hypoxia chamber continuously flushed with 5% CO_2_ and 94% N_2_. Partial oxygen pressure was monitored during incubation using a CO_2_/O_2_ gas controller (BioSpherix, 37 °C, 95% humidity).

### 4.5. Measurement of Oxygen Partial Pressure in Culture Media

The oxygen tension in the cell-free media incubated for 24 h (time of pre-conditioning before the addition to the cells) and 6 days (time of the full experiment) under normoxic or hypoxic conditions was measured using an ABL90 FLEX analyzer (Radiometer Medical, Denmark). After incubation, the medium was aspirated inside a laminar flow hood in atmospheric air or inside a hypoxia processing chamber using a hermetically sealable syringe arterial blood sampler (safePICO Aspirator). Then, samples of the medium from normoxic or hypoxc conditions were assayed in the ABL90 FLEX analyzer within 35 s, without exposure to air. The values of pO_2_ and pCO_2_ were expressed in torr, which was later calculated into mmHg (1 torr = 1 mmHg).

### 4.6. Assessment of Apoptosis

The PE Annexin V Apoptosis Detection Kit (Becton Dickinson) was used to detect apoptosis in cells treated with CisPt at IC_25_ dose (5.9 µM), Res at IC_25_ dose (63 µM), and CisPt+Res (at the same concentrations) in normoxia and hypoxia, as described in Section 4.4: Establishment of Hypoxic Culture Conditions and Experimental Outline. After 48 h incubation with drugs, cells were harvested and stained according to the manufacturer’s protocol. Then, the samples were analyzed with a FACSCalibur flow cytometer (Becton Dickinson), equipped with 355 nm, 405 nm, 488 nm, and 640 nm lasers. Data were analyzed using FCS Express 4 Flow software.

### 4.7. Cell Cycle Analysis

A2780 cells were seeded in 6-well plates at 7 × 10^4^ per well and then treated with CisPt, Res, and CisPt+Res in normoxia and hypoxia as described above. After a 48 h incubation with drugs under normoxic and hypoxic conditions, the cells were fixed in 70% ethanol, and stored at 4 °C overnight. Analysis of cell cycle was performed by flow cytometry after staining by propidium iodide (Becton Dickinson) for DNA content using a BD FACSCalibur flow cytometer (Becton Dickinson), equipped with 488 nm and 633 nm lasers. Cell fractions in G0/G1, S, and G2/M phases were calculated with Modfit software (Verity Software House, Topsham, ME, USA).

### 4.8. Immunofluorescence

A2780 cells were seeded in an 8-well Nunc Lab-Tek II Chamber Slide system (Thermo Fisher Scientific) at 5 × 10^3^/well and treated with CisPt, Res, and CisPt+Res in normoxia and hypoxia, as described above. Next, cells were fixed with 4% (*w*/*v*) paraformaldehyde in PBS for 10 min, permeabilized with 0.1% Triton X-100 in PBS for 20 min, blocked with PBS/0.01% Triton X-100 containing 5% goat serum for 1 h at RT, and incubated overnight at 4 °C with a primary mouse monoclonal anti-human Ki-67 antibody (isotype IgG_1_, kappa; diluted 1:200 in blocking solution; Agilent Dako, Santa Clara, CA, USA) in a humidified chamber. The binding of the antibody was detected using the secondary Alexa-Fluor 555 (AF-555)-labeled anti-mouse antibody (isotype IgG; 1:100, Thermo Fisher Scientific) for 1 h at RT. Cell nuclei were stained with bisbenzimide H33342 trichloride (Sigma-Aldrich) in PBS for 2 min at RT. Images were acquired with a fluorescence, Zeiss Axio Observer microscope at 40× magnification and analyzed using Z1/7—software Zen v.2.6 blue edition (Zeiss, Oberkochen, Germany). Negative controls omitted the primary antibody. The Ki-67-positive cells were quantified using ImageJ software. The experiments were performed in three biological repetitions.

### 4.9. Measurement of Reactive Oxygen Species (ROS)

Intracellular ROS levels were quantified using a fluorescent probe of 2′,7′-dichlorofluorescein diacetate (DCFDA, Sigma-Aldrich). Briefly, A2780 cells were seeded into 6-well plates at the 7 × 10^4^ density and were treated with CisPt, Res, and CisPt+Res for 1 h and 48 h in normoxia and hypoxia as described above. Then, cells were harvested and stained with 5 μM DCF-DA for 30 min at 37 °C. Next, they were washed with PBS and the signal was measured using a CytoFLEX flow cytometer (Beckman Coulter, Brea, CA, USA), using the excitation at 488 nm and emission at 780 nm. Analysis was performed using CytExpert software v.2.3.0.84 (Becton Dickinson). The mean MFI of intracellular ROS was normalized to the normoxia control as 1. Experiments were performed in triplicates.

### 4.10. Isolation of RNA and Quantitative PCR

Total RNA was extracted from the samples incubated under normoxia or hypoxia using the RNeasy Mini Kit (Qiagen, Hilden, Germany), and purified according to the manufacturer’s protocol. Two micrograms of RNA were reverse transcribed into cDNA using QuantiTect Reverse Transcription Kit (Qiagen), according to the manufacturer’s instructions. Samples were then subjected to real-time quantitative PCR using Light Cycler 480 (Roche). The relative abundance of target mRNA was calculated according to the ΔΔ cycle threshold method (ΔΔCt). mRNA expression levels of PPIA and GUSB were used as an internal control to normalize each qPCR reaction. The relative expression levels were calculated as fold enrichment of treated cells over the control cells. Following TaqMan probes (Thermo Fisher Scientific) used in RT-qPCR experiments: HIF-1 (Hs00153153_m1), PPIA (Hs01565699_g1), GUSB (Hs00939627_m1). The experiment was performed in three biological repetitions.

### 4.11. Enzyme-Linked Immunosorbent Assay

A2780 cells were incubated in normoxic or hypoxic conditions with CisPt, Res, and CisPt+Res for 48 h as described above. Subsequently, the medium was harvested, centrifuged (1000× *g*, 15 min., 4 °C), collected, and stored at −80 °C. The concentration of VEGF in media was measured by a Human VEGF165 Standard ABTS ELISA Development Kit according to the manufacturer’s protocol (PeproTech, London, UK). Absorbance was measured at 450 nm and protein levels were interpolated from the standard curve. Results were normalized to the normoxia control taken as 1. All standards and samples were measured in triplicate.

### 4.12. Western Blot Analysis

A2780 cells were lysed with RIPA buffer (Thermo Fisher Scientific), containing Protease Inhibitors Cocktail (Sigma-Aldrich), and frozen overnight. After thawing, the protein concentration was determined by the BCA protein assay kit (Thermo Fisher Scientific). Proteins (the same amount per well) were separated on 15% or 12% SDS-PAGE gel and transferred to nitrocellulose membrane (Bio-Rad, Feldkirchen, Germany), which was then blocked with 5% skim milk in Tris-buffered saline/Tween for 2 h at RT. After blocking, membranes were incubated overnight at 4 °C with: anti-cleaved caspase-3 (5A1E), anti-cleaved caspase-7 (D6H1), anti-caspase-3 (8G10), anti-caspase-7 (D2Q3L), anti-BAX (D2E11), anti-β-catenin (D10A8), anti-ZEB1 (D80D3), anti-vimentin (D21H3), anti-ZO-1 (all from Cell Signaling Technology, Danvers, MA, USA; isotype IgG), anti-HIF-1α (28b), anti-vinculin (V284), anti-SNAI1 (G-7), and anti-SNAI2 (A-7) antibodies (from Santa Cruz Biotechnology, Dallas, TX, USA; isotype IgG_1_ kappa), followed by incubation with horse anti-mouse or goat anti-rabbit IgG secondary antibodies conjugated with horseradish peroxidase (1:10.000; Vector Laboratories, Burlingame, CA, USA) for 1 h at RT. Signals were detected by enhanced chemiluminescence substrate (Santa Cruz Biotechnology) using X-ray films (Carestream, Rochester, NY, USA). The band intensity was quantified using ImageJ software. Band intensities were normalized to the intensities of their corresponding loading controls (Vinculin) and relative fold changes were calculated after normalization to the normoxia control as 1. Experiments were performed in triplicates.

### 4.13. Cell Migration Assay

A2780 cells were seeded in a 24-well plate at 4 × 10^3^ cells per well and were treated with CisPt, Res, and CisPt+Res in normoxia and hypoxia, as described above. The cell migration was recorded for 48 h at 20 min intervals at 5× magnification using a Zeiss Axio Observer microscope Z1/7—software Zen v.2.6 blue edition (Zeiss, Oberkochen, Germany). The velocity of cells was analyzed using the previously described method developed at the Jagiellonian University (Cracow, Poland) by the Cell Biology Department of the Faculty of Biology Biophysics and Biotechnology [90,91,92,93]. The movement of 50 single non-proliferating cells treated with CisPt or/and Res or the control cells under normoxic and hypoxic conditions was analyzed. The designated time points (5 h, 48 h) represent the average speed of cell movement in 5 h, e.g., a 48-h time point is 5 h between 45 and 50 h. The experiment was performed in triplicates (n = 150).

### 4.14. Data Analysis

Data were expressed as means ± SEM (standard error of the mean) for at least three biological experiments. To calculate IC_50_ values of CisPt and Res alone or in their combination, the combination index (CI) values from CalcuSyn software (BioSoft, v. 2.0) were used. Assessment of the normality of the distributions of the data was evaluated using the Shapiro–Wilk test. To estimate the effects of CisPt and Res alone and in combination on changes in IC_50_ values, two-tailed Student’s *t* and Mann–Whitney U tests were used. For comparison between normoxia- and hypoxia-treatment groups, GraphPad Prism software v. 9.0 (GraphPad Software, San Diego, CA, USA) was used, using one-way ANOVA or two-way ANOVA followed by a Tukey’s post hoc test (for Gaussian distribution). For non-Gaussian distribution, the non-parametric Mann–Whitney U test or Kruskal–Wallis was used, followed by a Dunn’s post hoc test. *p* values less than 0.05 were considered statistically significant.

## 5. Conclusions

Our study is the first demonstration of the effects of the combination of Res with CisPt in A2780 OC cells under hypoxic conditions. We show that the combined CisPt+Res treatment of A2780 cells under hypoxic conditions imitating the solid tumor oxygen niche does not improve the desired therapeutic effects of CisPt treatment. Therefore, it potentially discourages the use of Res as an adjuvant for CisPt anticancer therapy, and certainly requires further studies. Moreover, the increased migration and the EMT-like phenotype of A2780 cells observed by us under hypoxic conditions might be associated with the enhanced selection of cancer cells to a more aggressive phenotype. Our work indicates that anticancer activities of drugs should be tested in conditions that report as much as possible the real tumor microenvironment. Such tests should especially take into account hypoxic stress [94], which is a condition allowing an equilibrated extrapolation and potential applications to translational personalized medicine. We are convinced that the application of hypoxia as a key parameter for in vitro assays for the usefulness of potential anticancer drugs will provide a less biased assessment for future anticancer treatments.

## Figures and Tables

**Table 1:** The values of oxygen pressure and carbon dioxide pressure in the culture media incubated for 24 h and 144 h (6 days) under normoxic and hypoxic conditions.

	Normoxia		Hypoxia	
	24 h	144 h (6 Days)	24 h	144 h (6 Days)
**pO_2_**	146.8 ± 1.7 mmHg	146.3 ± 1.7 mmHg	14.7 ± 2.7 mmHg	15.2 ± 0.3 mmHg
**pCO_2_**	36.7 ± 4.6 mmHg	39 ± 1.5 mmHg	31.9 ± 0.1 mmHg	32.9 ± 0.5 mmHg

## Data Availability

Not applicable.

## Supplementary Materials

The following supporting information can be downloaded at: https://www.mdpi.com/article/10.3390/ijms24065715/s1. Figure S1: A model function *f*(*x*) that is approximated by a linear function (upper fragments of trapezoids) and how the trapezoidal rule utilizes a sequence of sample trapezoids; Figure S2: The synergistic analysis of cisplatin in the combination with resveratrol for five ovarian carcinoma cell lines; Figure S3: Effect of hypoxia and cisplatin, resveratrol, and their combination on caspase-3 and caspase-7; Figure S4: The morphology changes in A2780 cells after treatment with cisplatin, resveratrol, and their combination under normoxic and hypoxic conditions; Table S1: Human OC cell line characteristics; Table S2: Survival curves of human ovarian carcinoma cell lines reported on the numerical detailed comparable AUC (area under the curve) values in a.u.; Table S3: Dose-effect and median-effect analyses based on the calculations with the use of CalcuSyn software performed in five ovarian carcinoma cell lines. References [95,96,97,98,99,100,101,102,103,104,105] are cited in Supplementary Materials.
