# Supplementary material for: Hypoxia, but Not Normoxia, Reduces Effects of Resveratrol on Cisplatin Treatment in A2780 Ovarian Cancer Cells: A Challenge for Resveratrol Use in Anticancer Adjuvant Cisplatin Therapy
Source: Int J Mol Sci. 2023 Mar 16;24(6):5715. doi: 10.3390/ijms24065715 (PMC10051682; doi:10.3390/ijms24065715)
Supplement: Supplementary file 1 [file ijms-24-05715-s001.zip › ijms-2194490-supplementary.pdf]

International Journal of *Molecular Sciences*

MDPI

Supplementary Information

Hypoxia, but Not Normoxia, Reduces Effects of Resveratrol on Cisplatin Treatment in A2780 Ovarian Cancer Cells: A Challenge for Resveratrol Use in Anticancer Adjuvant Cisplatin Therapy

Agnieszka Synowiec[1,*], Klaudia Brodaczewska[1#], Gabriel Wcisło[2,3#], Aleksandra Majewska[1,4], Agata Borkowska[1,4], Aleksandra Filipiak-Duliban[1,4], Aleksandra Gawrylak[1,5], Kinga Wilkus[1,4], Katarzyna Piwocka[6], Agata Kominek[6], Halina Waś[1], Sławomir Lewicki[7], Jacek Siewiera[8], Cezary Szczylik[2,3], Jolanta Szenajch[1], Jacek Z. Kubiak[1,9] and Claudine Kieda[1,10]

Methods

Survival curve and trapezoidal method

Survival curves were depicted on figures in which y-axis is the percent growth control in relation to proper doses of given drugs alone or in combination placed on x-axis. The curves represent the function of both variables, i.e. the percent growth control and used doses of cisplatin alone or its combination with resveratrol. Such functions can be analyzed by using the numerical integration. The latter term embraces a broad set of algorithms for calculating the numerical value of a definite integral and the numerical solution of differential equations. We focused on calculating the numerical value of the area under the curve (AUC) mapping the survival of each ovarian carcinoma cell line treated with cisplatin alone or its combination with resveratrol in a given protocol of therapy.

The integration is evaluated with the use a finite set of points called *integration points* and the sum of these values is utilized to approximate the integral. The approximation error is in fact a function of the number of integrated evaluations dependent on a set of integration points. Intuitively, a method that can offer a small error for a small number of evaluations is able to be defined optimal. By reducing the number of evaluations of integration one can get reduced the total round-off error [95].

Numerical quadrature rules can be obtained by calculating *interpolating function* which in turn is easy to integrate. Practically, these interpolating functions are polynomials of very high degree, oscillating broadly, or of low degree usually linear and quadratic, more often used in calculations. The simplest method of interpolating function is called *rectangle rule*. Numerical quadrature is a synonym of the term of *numerical integration* which approximates the integral by finite sums of the interval of integration $[a, b]$ based on proper integration points. We decided to compute the integral (I):

$$I = \int_a^b f(x)dx \tag{1}$$

for which we generally assume that the functions $f(x)$ are Riemann integrable [96].

The basic method relies on approximating the integral I which is obtained by the limiting process

$$I = \int_a^b f(x)dx = \lim_{n\to\infty} \frac{b-a}{n} \sum_{k=1}^{n} f(x_k^o) \tag{2}$$

for which $x_o = a, x_n = b$, and for which the value $I$is independent of the point $x_k^o \in [x_{k-1}, x_k]$. But beyond our initial assumption, not all functions $f(x)$ fulfill requirements to be Riemann integrable. This problem is able to be ignored by approximation I according to the simpler formula

$$I \approx \frac{b-a}{n} \sum_{k=1}^{n} f(x_k^o) \quad (3)$$

which is responsible for approximation based upon so called *the rectangular rule*. The constant interpolation of $f(x)$ is provided with fitting a constant polynomial on some interval

$$h = \frac{b-a}{n} = x_k - x_{k-1} \quad (4)$$

The interpolating function very often is measured by a straight line passing through the integration points and this method is based upon the *trapezoidal rule*. We make an accurate approximation by dividing the interval $[a, b]$ in several n subintervals that are calculated and subsequently summing up to get the altogether results. This method is called a *composite rule*. Numerical integration with the use of trapezoidal rule is based on the linear interpolation of the function on all compartments $[x_{k-1}, x_k]$ and the approximation of $\int_{x_{k-1}}^{x_k} f(x)dx$ is given by the area of the trapezoid calculated

$$\int_{x_{k-1}}^{x_k} f(x)dx \approx \frac{1}{2}[f(x_k) + f(x_{k-1})](x_k - x_{k-1}) = \frac{1}{2}[f(x_{k-1}) + f(x_k)]h \quad (5).$$

Adopting the formula (4) to the trapezoidal rule one can use the formula (5) after applying (4) for $k = 1$ to $k = n$ to obtain

$$\int_a^b f(x)dx \approx \frac{h}{2} \sum_{k=1}^{n} [f(x_{k-1}) + f(x_k)] = T(n) \quad (6)$$

and the adding up extends to

$$T(n) = \frac{h}{2}[f(x_0) + 2f(x_1) + 2f(x_2) + \cdots + 2f(x_{n-1}) + f(x_n)] \quad (7)$$

where $n \in N$ (set of natural numbers) [97,98].

The Figure S1 shows a model function $f(x)$ which is approximated by a linear function and how trapezoidal rule utilizes a sequence of sample trapezoids.

Survival curves were obtained by measuring absorbance at 570 nm and using 600 nm as a reference weave-length after adding AlamarBlue reagent as 10% (typically, 20 µl) of the sample volume, in practice a cell culture well with sample volume of 200 µl. These curves were depicted in relation to each dose of used cisplatin (CisPt) alone as the reference (control) value or the combination of CisPt and resveratrol (Res). We typically measure eight or nine such points defined as integration points that make possible to compute the surface area each trapezoid following their summation to obtain a final result for each curve. Practically, it is easy to perform the calculations using the formula

$$T(n) = \frac{h}{2}[f(x_0) + 2f(x_1) + 2f(x_2) + \cdots + 2f(x_{k-1}) + 2f(x_k) + 2f(x_{k+1}) + \cdots + 2f(x_{n-1}) + f(x_n)] (8)$$

that is strictly linked to the visualized model function $f(x)$ presented in the Figure S1. Technically, it is enough to adjust one's data from the dataset, e.g. Excel dataset, and to use the formula (8), more precise than the formula (7), and ascribed to the function presented in Figure S1, for generating own results. Having a value of surface each survival curve, the statistical comparison among of them is easy to compute in order to show the real difference being significant or not significant.

Dose-effect, median-effect analyses and combination activity

To determine the combined activity of CisPt and Res, we used the dose-effect analysis and median-effect analysis (9). The median effect equation states that:

$$\frac{f_a}{f_u} = (\frac{D_x}{D_m})^m \ and \ f_a + f_u = 1, so \ \ f_u = 1 - f_a) \tag{9}$$

where f_a is the affected fraction of cells by a drug and f_u is the unaffected fraction of cells in an experimental system; D_x is the dose of a drug, D_m is the median-effect dose defined also IC_{50} , m is the the exponent of the slope of the median-effect plot. The magnitude of the m value changes with the sigmoidicity of the dose-effect curve. A plot of $y = log\ (\frac{f_a}{f_u})$ with respect to $x = logD_x$ is to be linear having a slope of m, D_x can be calculated from the equation (10):

$$D_x = D_m[\frac{f_a}{1 - f_a}]^{1/m} \tag{5}$$

Combination index (CI) was calculated based on the data from three independent experiments each embracing at least three points of measured values for each drug separately and for their combination. The equation (11) for CI states:

$$CI = \frac{D_1}{D_{1x}} + \frac{D_2}{D_{2x}} \tag{6}$$

D_1 and D_2 represent the concentration of drug 1 (CisPt) and drug 2 (Res) used in combination to get the percent of inhibition whereas D_{1x} and D_{2x} are the concentrations of drugs 1 and 2 to achieve the percent of inhibition when utilized alone. Such a quantitative analysis of both combined drugs determines if the drug combinations are additive, synergistic or antagonistic with values of CI = 1, CI < 1 and CI > 1, respectively. To calculate all variables analyzed dealing with combination action of CisPt and Res we used purchased full version of CalcuSyn for Windows software for dose effect analysis Version 2.0 (Biosoft, Cambridge, UK). Chou-Talalay isobologram method is graphical illustration pharmacological interactions between CisPt and Res as a straight line connecting points the affected fraction of cells by a drug (f_a) against the constant ratio mixture of drug 1 and drug 2 on the x-axes and y-axes [89]. The equation (11) for CI is equivalent to the equation for the sum of the fractional inhibitory concentration (∑FIC), when fractional inhibitory concentration (FIC) was calculated by dividing the concentration of CisPt or Res needed to obtain the IC_{50} when used in a mixture by the concentration of CisPt or Res needed to obtain the IC_{50} when used alone.

To investigated the combinatory effect of CisPt with Res we used CI at ED50 (50% effect doses), ED75 (75% effect doses) and ED90 (90% effect doses).

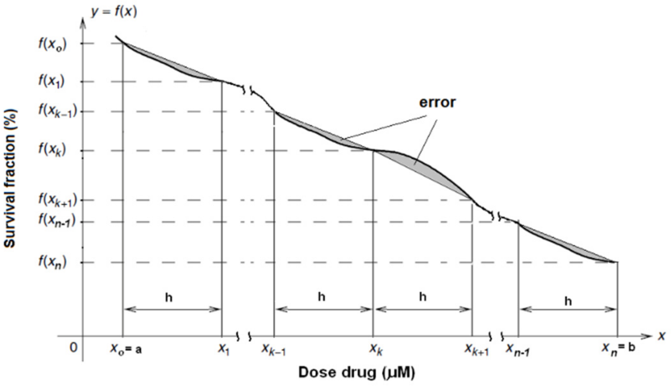

Figure S1. A model function $f(x)$ which is approximated by a linear function (upper fragments of trapezoids) and how the trapezoidal rule utilizes a sequence of sample trapezoids.

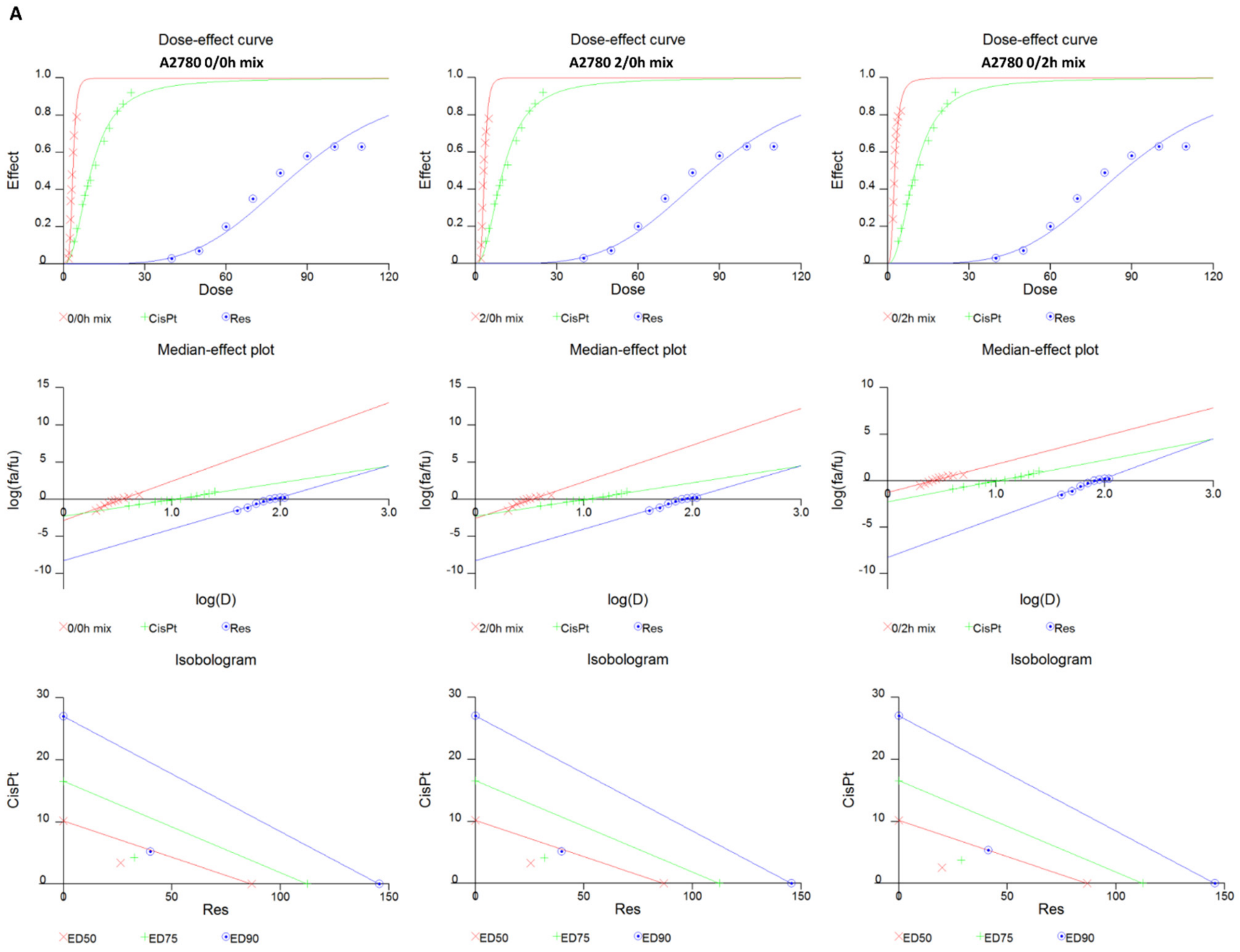

B

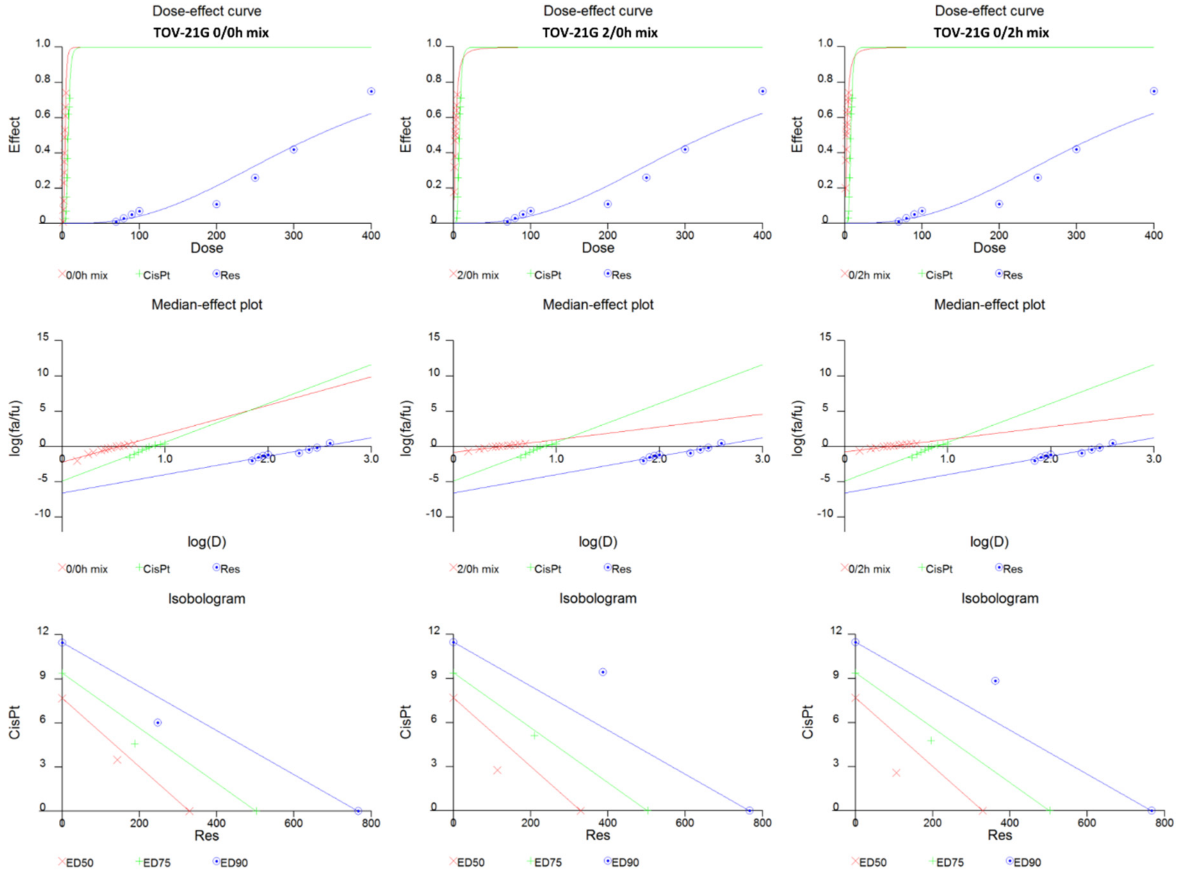
Dose-effect curve
TOV-21G 0/0h mix
TOV-21G 2/0h mix
TOV-21G 0/2h mix
Effect
Dose
0/0h mix
2/0h mix
0/2h mix
CisPt
Res
Median-effect plot
log(fa/fu)
log(D)
Isobologram
CisPt
Res
ED50
ED75
ED90

C

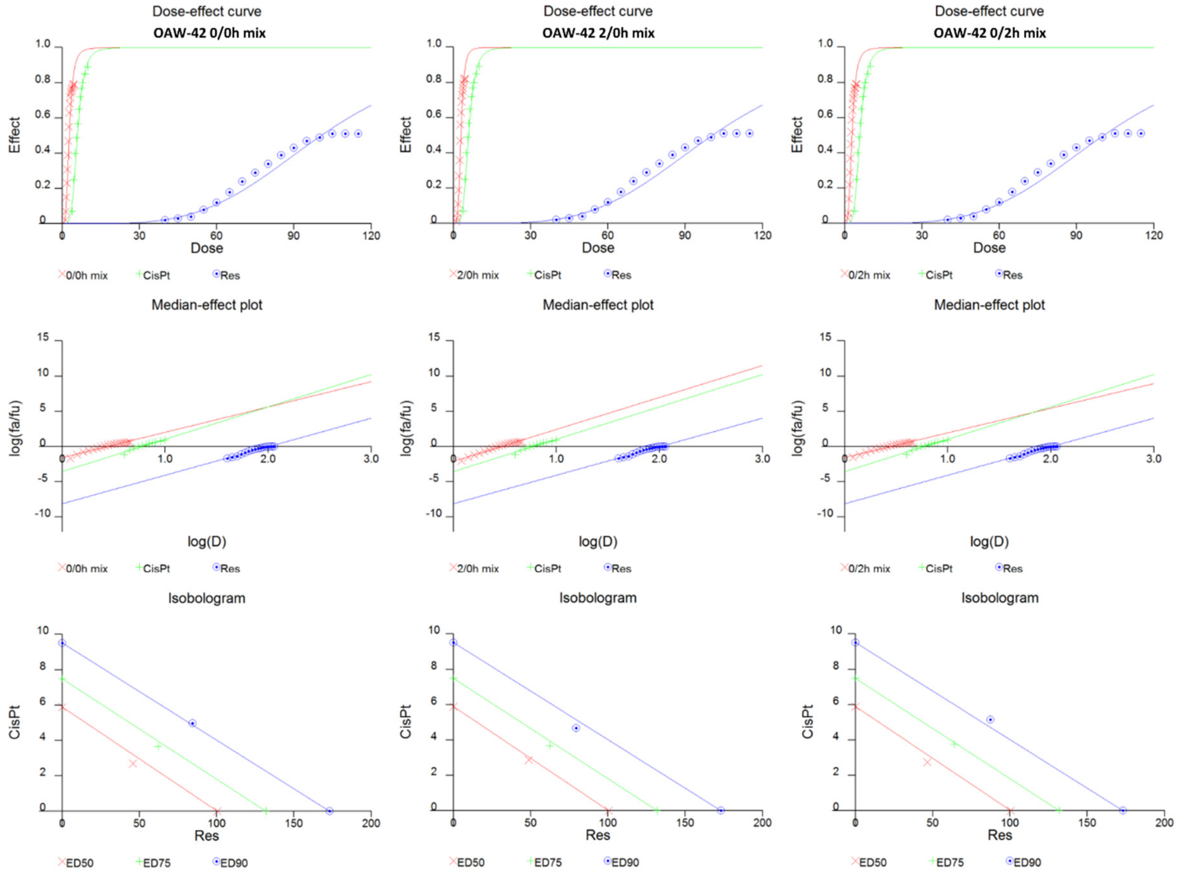
Dose-effect curve
OAW-42 0/0h mix
OAW-42 2/0h mix
OAW-42 0/2h mix
Effect
Dose
0/0h mix
2/0h mix
0/2h mix
CisPt
Res
Median-effect plot
log(fa/fu)
log(D)
Isobologram
CisPt
Res
ED50
ED75
ED90

D

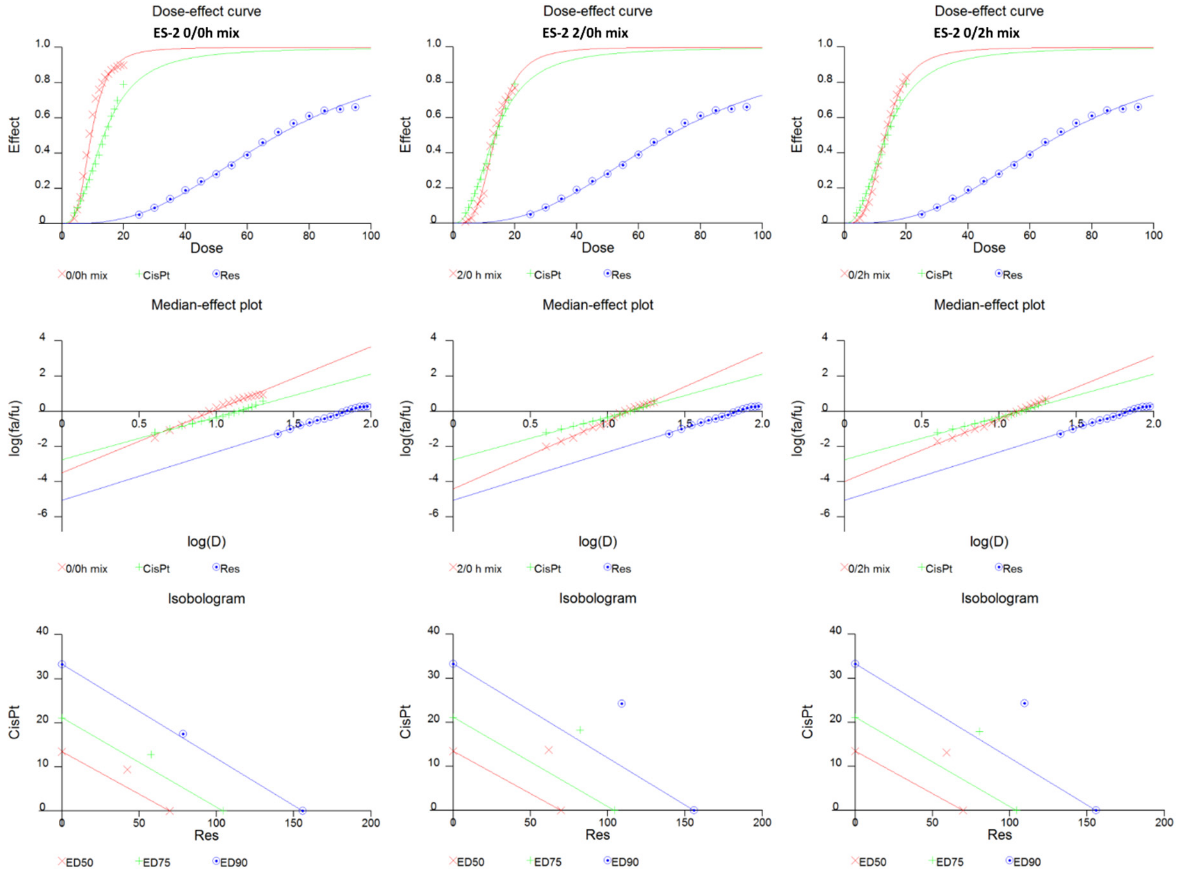
Dose-effect curve
ES-2 0/0h mix
ES-2 2/0h mix
ES-2 0/2h mix
Effect
Dose
0/0h mix
2/0 h mix
0/2h mix
CisPt
Res
Median-effect plot
log(fa/fu)
log(D)
Isobologram
CisPt
Res
ED50
ED75
ED90

E

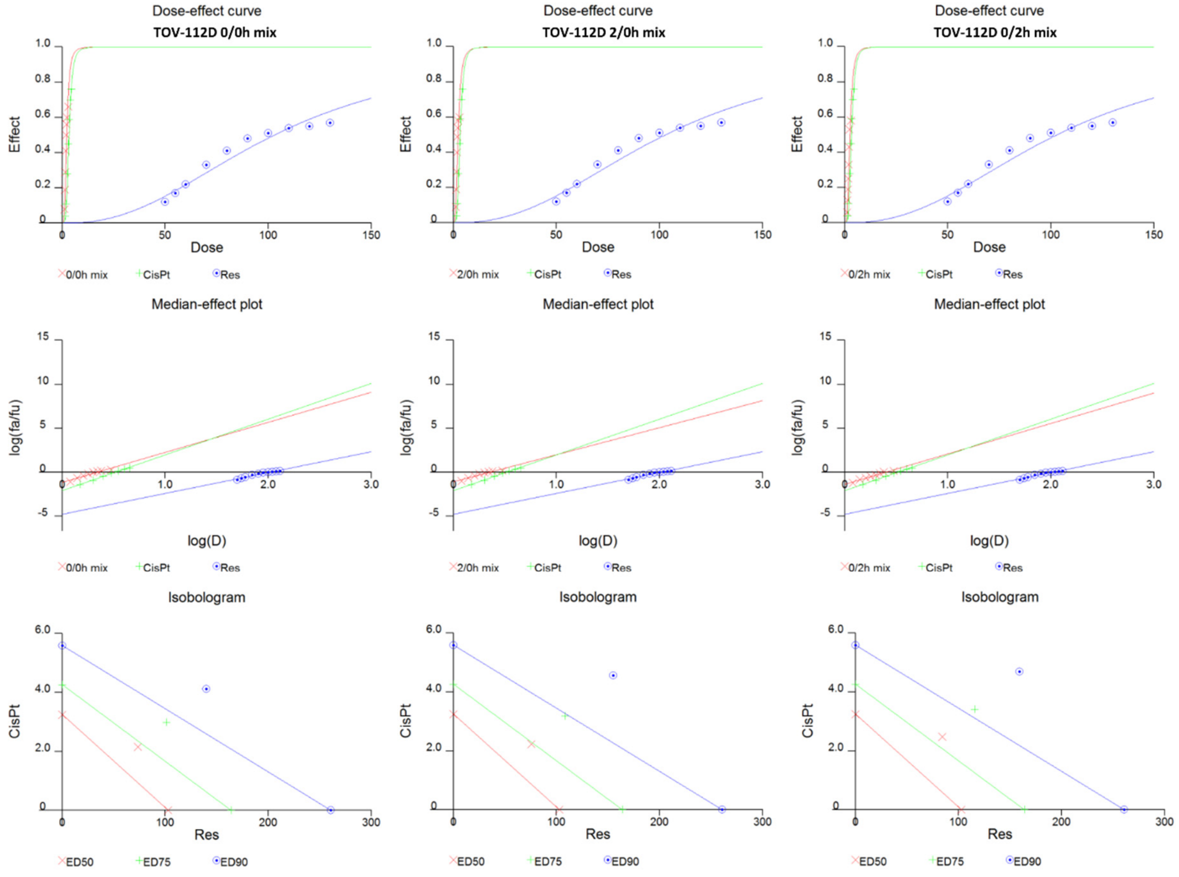
Dose-effect curve
TOV-112D 0/0h mix
TOV-112D 2/0h mix
TOV-112D 0/2h mix
Effect
Dose
0/0h mix
2/0h mix
0/2h mix
CisPt
Res
Median-effect plot
log(fa/fu)
log(D)
Isobologram
CisPt
Res
ED50
ED75
ED90

Figure S2. The synergistic analysis of cisplatin in the combination with resveratrol for five ovarian carcinoma cell lines.

CalcuSyn software v.2.0 based analysis of synergistic effect between cisplatin (CisPt) and resveratrol (Res) for following ovarian cancer cell lines: **(A)** A2780; **(B)** TOV-21G; **(C)** OAW-42; **(D)** ES-2; **(E)** TOV-112D expressed in the form: dose-effect curves (upper panel), median-effect plots (middle panel) and isobologram plots (lower panel) as a graphical presentation of combination index (CI) at ED50, ED75 and ED90 effect levels of inhibition cells. The individual doses of CisPt and Res to achieve 50% (red line), 75% (green line), and 90% (blue line) growth inhibition were plotted on the x- and y-axes. Data points on the isobolograms, corresponding to the CI values, located above the straight line indicate antagonism between CisPt and Res; on the line indicate additive effect, and below the line indicate synergism between these drugs. Red x symbol, green plus sign and blue open dotted circle indicates the CI values at ED50, ED75 and ED90, respectively. Ovarian cancer cell lines were treated with CisPt or Res alone and in their combination (at a constant ratios IC_{50}'s values of CisPt and Res alone) in the following combinations: both compounds were added at the same time (mix 0/0 h), Res given 2 hours before CisPt (mix 2/0 h) or given 2 hours after CisPt (mix 0/2 h). Data are representative of three experiments.

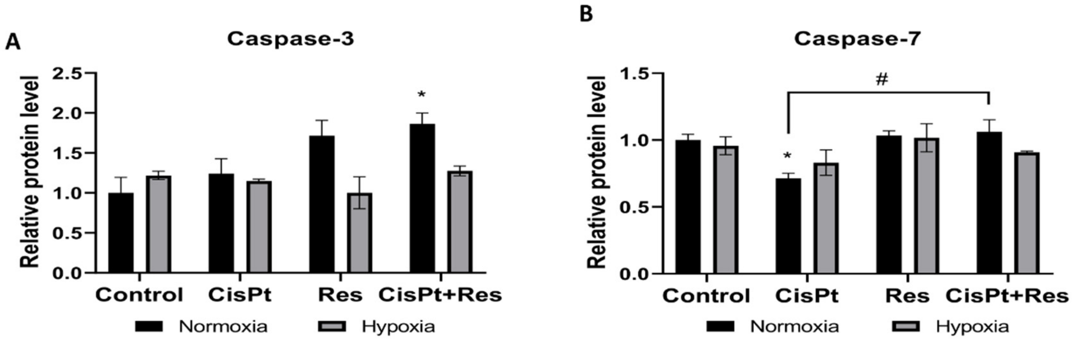

Figure S3. Effect of hypoxia and cisplatin, resveratrol, and in their combination on caspase-3 and caspase-7.

The A2780 cells were seeded in 6-well plates $7x10^4$ per well and cultured for 24 h, then treated under normoxia or hypoxia for 72 h. Next, the cells were treated with a vehicle or IC_{25} doses of cisplatin (CisPt) alone, resveratrol (Res) alone and in the combination (CisPt+Res) for 48 h under normoxia or hypoxia. Apoptosis was assayed by Annexin V apoptosis detection kit followed by flow cytometry analysis. Quantified data of Western blot analysis **(A)** of caspase-3; **(B)** of caspase-7. The whole-cell extracts were prepared, separated on sodium dodecyl sulphate–polyacrylamide gel electrophoresis (SDS-PAGE), and subjected to Western blot using specific antibodies. The graphs represent the densitometric analysis of the immunoblots. Vinculin was used as loading control and normalized to normoxia control as 1. The results shown are mean ±SEM of three independent experiments. $^*p<0.05$ compared to normoxia control (black asterix); $^{\#}p<0.05$ CisPt in normoxia compared to CisPt in combination with Res in normoxia as determined by one-way ANOVA and two-way ANOVA followed by *Tukey's post-hoc* test were taken as significant.

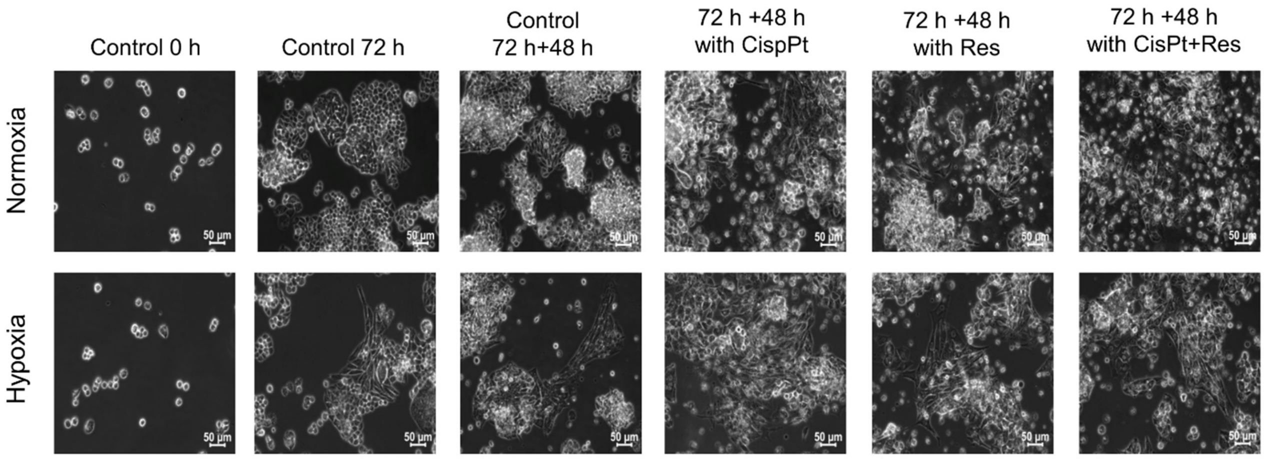

Figure S4. The morphology changes in A2780 cells after treatment with cisplatin, resveratrol, and in their combination under normoxic and hypoxic conditions.

The A2780 cells were treated under normoxia (upper panel) and hypoxia (lower panel) for 72 h and next, the cells were treated with a vehicle or IC_{25} doses of cisplatin (CisPt), resveratrol (Res) and CisPt+Res for 48 h under normoxia or hypoxia. The cells were observed under 20×magnification, scale bar 50 µm.

Table S1. Human ovarian cancer cell line characteristics.

Cell line.	Histopathology	Origin	Treatment before isolation	Biological and molecular characterization	References
TOV-21G	Adenocarcinoma/clear cell carcinoma	Primary tumor	Untreated	• DT:1.5<br>• P53: wild type<br>• CDKN2A: wild type<br>• TGFbetaRII: mutation in exon 3<br>• Microsatellite instability: plus<br>• Fraction genome altered: 0.05<br>• Mutations per Mb: 13.42<br>• Cisplatin sensitive<br>• FANCD2 mono-Ub: minus<br>• FANCF mRNA expression: minus<br>• MSP methylated/unmethylated	[99-101]
TOV-112D	Adenocarcinoma/endometrioid carcinoma	Primary tumor	Untreated	• DT: 0.8<br>• P53 mut. (Arg->His exon 6, codon 175)<br>• CDKN2A: wild type<br>• TGF betaRII: wild type<br>• Microsatellite instability: minus<br>• Cisplatin sensitive<br>• FANCD2 mono-Ub: plus<br>• FANCF mRNA expression: plus<br>• MSP unmethylated	[99,100]
OAW-42	Cystadenocarcinoma	Ascites	Cisplatin	• Fraction genome altered: 0.43<br>• Mutations per Mb: 3.28<br>• *icb-1* gene knocked down results upregulation oncogenes regulated by ERalpha and TNF	[100-103]

				• FANCD2 mono-Ub: plus<br>• FANCF mRNA expression: plus<br>• MSP methylated/unmethylated	
A2780	Unknown	Primary tumor	Untreated	• Fraction genome altered: 0.07<br>• Mutations per Mb: 4.59<br>• Cisplatin sensitive but become resistant over time<br>• Increased resistance to cytostatics after transfection with mutated p53<br>• FANCD2 mono-Ub: plus<br>• FANCF mRNA expression: plus<br>• MSP unmethylated	[100,101,103-105]
ES-2	Clear cell carcinoma	Primary tumor	Untreated	• Fraction genome altered: 0.28<br>• Mutations per Mb: 6.00<br>• P-glycoprotein. MDR 170 (low expression)<br>• FANCD2 mono-Ub: plus<br>• FANCF mRNA expression: plus<br>• MSP unmethylated<br>• Exhibit resistance to cisplatin	[100,101]

Abbreviations: DT: doubling time in days; ER-alpha: estrogen receptor alpha; FANCD2: Fanconi Anemia Protein D2; FANCF: Fanconi Anemia Protein F; Mono-U: mono-ubiquitination; MSP: methylation-specific PCR; salinomycin: antibiotic isolated from *Streptomyces albus.*

Table S2. Survival curves of human ovarian carcinoma cell lines reported on the numerical detailed comparable AUC (area under the curve) values in a.u.

	**TOV-21G**						**Constant ratio (IC$_{50}$'s ratio) CisPt:Res = 1:43**						
	**CisPt**	**Mix 0/0h (a)**	**t**	**p**	**Mix 2/0h (b)**	**t**	**p**	**Mix 0/2h (c)**	**t**	**p**	**Mix mean effect**	t	p
AUC [a.u.]	743.13 ±12.89 (711.08-775.17)	403.47±12.42 (372.61-434.33)	32.85	<0.001	365.06±10.72 (338.41-391.7)	39.03	<0.001	345.4±6 (330.49-360.31)	48.41	<0.001	371.31±29.53 (297.94-444.67)	19.98	<0.001
Difference between Mix protocols						(a) vs (b) 4.05	(a) vs (b) 0.015		(a) vs (c) 7.28 (b) vs (c) 2.76	(a) vs (c) 0.001 (b) vs (c) NS			
	TOV-112D						Constant ratio (IC$_{50}$'s ratio) CisPt:Res = 1:32						
	CisPt	Mix 0/0h (a)	t	p	Mix 2/0h (b)	t	p	Mix 0/2h (c)	t	p	Mix mean effect	t	p
AUC [a.u.]	285.31 ±16.91 (243.29-327.33)	233.92±3.32 (225.67-242.17)	5.16	0.006	255.33±9.92 (230.68-279.97)	2.64	NS	265.76±14.92 (228.68-302.83)	1.5	NS	251.67±16.23 (211.34-291.99)	2.48	NS
Difference between Mix protocols						(a) vs (b) 3.54	(a) vs (b) 0.023		(a) vs (c) 3.6 (b) vs (c) 1.0	(a) vs (c) 0.022 (b) vs (c) NS			
	OAW-42						Constant ratio (IC$_{50}$'s ratio) CisPt:Res = 1:17						

	CisPt	Mix 0/0h (a)	t	p	Mix 2/0h (b)	t	p	Mix 0/2h (c)	t	p	Mix mean effect	t	p
AUC [a.u.]	496.93 ±16.91 (489.48-504.38)	242.87±10.06 (217.85-267.88)	41.88	<0.001	254.69±7.64 (235.69-273.68)	51.07	<0.001	245.47±5.12 (232.74-258.19)	73.38	<0.001	247.67±6.21 (232.24-263.1)	62.59	<0.001
Difference between Mix protocols						(a) vs (b) 1.61	(a) vs (b) NS		(a) vs (c) 0.39 (b) vs (c) 1.73	(a) vs (c) NS (b) vs (c) NS			
A2780								Constant ratio (IC_{50}'s ratio) CisPt:Res = 1:8.7					
	CisPt	Mix 0/0h (a)	t	p	Mix 2/0h (b)	t	p	Mix 0/2h (c)	t	p	Mix mean effect	t	p
AUC [a.u.]	956.58 ±41.42 (853.67-1059.49)	386.6±6.21 (371.16-402.03)	23.56	<0.001	383.79±49.39 (261.09-506.49)	15.38	<0.001	339.63±81.07 (138.22-541.03)	11.73	<0.001	370.0±26.34 (304.56-43544)	20.69	<0.001
Difference between Mix protocols						(a) vs (b) 0.92	(a) vs (b) NS		(a) vs (c) 1.0 (b) vs (c) 0.8	(a) vs (c) NS (b) vs (c) NS			
ES-2								Constant ratio (IC_{50}'s ratio) CisPt:Res = 1:5.5					
	CisPt	Mix 0/0h (a)	t	p	Mix 2/0h (b)	t	p	Mix 0/2h (c)	t	p	Mix mean effect	t	p

AUC [a.u.]	1287.45 ±51.76 (1158.87 - 1416.03)	914.36±29.56 (840.91-987.81)	10.84	<0.001	1285.94 ±29.5 (1212.64 - 1359.23)	0.044	NS	1241.54 ±6.88 (1224.44 - 1258.63)	1.52	NS	1147.28.± 202.93 (643.16-1651.39)	1.15	NS
Difference between Mix protocols						(a) vs (b) 15.4	(a) vs (b) <0.001		(a) vs (c) 18.66 (b) vs (c) 2.53	(a) vs (c) <0.001 (b) vs (c) NS			

Abbreviations: Data expressed in mean ± SD (standard deviation) and in (95%CI= confidence interval); t = t-statistics; p = p-value of difference statistically significant or not at p<0.05; mix 0/0 h=CisPt and Res added at the same time; mix 2/0 h= Res given 2 hours before CisPt; mix 0/2 h= Res given 2 hours after CisPt; AUC = area under curve; a.u. = arbitrary units; NS = statistically non-significant. P-values has been determined by unpaired T Student.

Table S3. Dose-effect and median-effect analyses based on the calculations with the use of CalcuSyn software performed in five ovarian carcinoma cell lines.

**A2780**								**Constant ratio (IC_{50}'s ratio) CisPt:Res = 1:8.7**	
	**CI at ED50**	**CI at ED75**	**CI at ED90**	**CisPt Dm (IC_{50})**	**Changes of IC_{50} CisPt in mixes *vs.* IC_{50} CisPt alone (fold)**	**p-value**	**Res Dm (IC_{50})**	**m**	**r**
CisPt (alone)				10.17±0.65 (8.56-11.78)				2.11±0.42 (1.06-3.16)	0.95±0.0(0.89-1.02)
Res (alone)							89.09±4.21 (78.63-99.56)	5.21±0.17 (4.79-5.63)	0.98±0.01 (0.95-1.01)

Mix 0/0h (a)	0.67±0.02 (0.61-0.74)	0.58±0.07 (0.40-0.76)	0.52±0.10 (0.27-0.76)	3.43±0.06 (3.29-3.58)	2.75±0.19 (2.28-3.21)	<0.001	29.87±0.52 (28.58-31.16)	5.42±1.13 (2.62-8.22)	0.98±0.01 (0.95-1.01)
Mix 2/0h (b)	0.65±0.03 (0.58-0.71)	0.56±0.07 (0.40-0.73)	0.51±0.09 (0.29-0.72)	3.30±0.13 (2.97-3.63)	3.07±0.23 (2.49-3.65)	<0.001	28.67±1.16 (25.80-31.54)	5.08±0.78 (3.13-7.02)	0.93±0.02 (0.87-0.99)
Mix 0/2 h (c)	0.51±0.03 (0.44-0.59)	0.51±0.07 (0.33-0.69)	0.53±0.11 (0.26-0.80)	2.60±0.11 (2.34-2.87)	3.62±0.14 (3.28-3.96)	<0.001	22.65±0.93 (20.35-24.96)	3.11±0.32 (2.3-3.91)	0.95±0.01 (0.92-0.98)
Mix median effect	0.61 ±0.09 (0.39-0.83)	0.55 ±0.04 (0.46-0.64)	0.52 ±0.01 (0.50-0.54)	3.11±0.45 (2.0-4.22)	3.15±0.44 (2.05-4.24)	<0.001	27.06±3.87 (17.45-36.67)	4.58±1.28 (1.41-7.75)	0.95±0.02 (0.91-0.98)
		**TOV-21G**					**Constant ratio (IC_{50}'s ratio) CisPt:Res = 1:43**		
	**CI at ED50**	**CI at ED75**	**CI at ED90**	**CisPt Dm (IC50)**	**Changes of IC_{50} CisPt in mixes *vs.* IC_{50} CisPt alone (fold)**	**p-value**	**Res Dm (IC50)**	**m**	**r**
CisPt				7.69±0.1 (7.45-7.93)				5.82±0.82 (3.81-7.86)	0.96±0.01 (0.93-0.99)
Res							329.97±9.9 (305.36-354.57)	2.67±0.22 (2.14-3.21)	0.96±0.01 (0.93-0.98)
Mix 0/0h (a)	0.91 ±0.03	0.91±0.01	0.91±0.04 (0.82-1.01)	3.52±0.02	2.21±0.05	<0.001	150.22±3.01	3.77±0.09	0.98±0.01

	(0.84-0.98)	(0.87-0.94)		(3.47-3.58)	(2.09-2.33)		(142.74-157.7)	(3.54-4.0)	(0.96-1.01)
Mix 2/0h (b)	0.74±0.07 (0.57-0.92)	0.89±0.02 (0.85-0.93)	1.08±0.06 (0.93-1.23)	2.93±0.15 (2.54-3.31)	2.71±0.23 (2.13-3.28)	<0.001	125.91±6.66 (109.36-142.27)	2.4±0.17 (1.98-2.820)	0.96±0.0 (0.94-0.97)
Mix 0/2h (c)	0.70±0.01 (0.67-0.74)	0.86±0.05 (0.74-0.97)	1.06±0.14 (0.73-1.40)	2.7±0.1 (2.44-2.96)	2.99±0.02 (2.94-3.03)	<0.001	115.93±4.49 (104.78-127.08)	2.3±0.54 (0.96-3.64)	0.96±0.04 (0.85-1.07)
Mix median effect	0.78±0.11 (0.51-1.06)	0.89±0.03 (0.82-0.95)	1.02±0.09 (0.79-1.25)	3.5±0.42 (2.0-4.10)	2.64±0.40 (1.66-3.62)	<0.001	130.69±17.64 (86.87-174.50)	2.82±0.82 (0.78-4.86)	0.97±0.01 (0.94-1.0)
			**OAW-42**					**Constant ratio (IC_{50}'s ratio) CisPt:Res = 1:17**	
	**CI at ED50**	**CI at ED75**	**CI at ED90**	**CisPt Dm (IC50)**	**Changes of IC_{50} CisPt in mixes *vs.* IC_{50} CisPt alone (fold)**	**p-value**	**Res Dm (IC50)**	**m**	**r**
CisPt (alone)				5.88±0.1 (5.63-6.13)				4.66±0.75 (2.78-6.53)	0.96±0.005 (0.94-0.97)
Res (alone)							99.44±4.11 (89.22-109.66)	4.41±0.9 (2.15-6.66)	0.97±0.01 (0.94-0.99)
Mix 0/0h (a)	0.92±0.04 (0.82-1.02)	0.97±0.02 (0.92-1.02)	1.02±0.03 (0.96-1.029	2.69±0.14 (2.33-3.06)	2.19±0.16 (1.80-2.57)	<0.001	45.91±2.46	3.68±0.42 (2.62-4.74)	0.96±0.01 (0.93-0.98)

							(39.78-52.03)		
Mix 2/0h (b)	0.99±0.04 (0.88-1.09)	0.98±0.03 (0.90-1.05)	0.97±0.09 (0.76-1.19)	2.88±0.1 (2.62-3.14)	2.04±0.06 (1.88-2.19)	<0.001	49.14±1.79 (44.69-53.58)	4.67±0.81 (2.64-6.7)	0.97±0.005 (0.95-0.98)
Mix 0/2h (c)	0.94±0.03 (0.85-1.03)	1.00±0.04 (0.90-1.09)	1.00±0.09 (0.90-1.09)	2.75±0.07 (2.57-2.92)	2.14±0.02 (2.09-2.19)	<0.001	46.83±1.96 (43.83-49.8)	3.58±0.49 (2.35-4.8)	0.96±0.01 (0.92-1.0)
Mix median effect	0.95±0.03 (0.87-1.03)	0.98±0.02 (0.94 - 1.02)	1.00±0.02 (0.94-1.06)	2.77±0.09 (2.53-3.01)	2.12±0.08 (1.93-2.31)	<0.001	47.29±1.66 (43.15-51.42)	3.97±0.6 (2.47-5.47)	0.96±0.005 (0.94-0.97)
			**ES-2**				**Constant ratio (IC_{50}'s ratio) CisPt:Res = 1:5.5**		
	**CI at ED50**	**CI at ED75**	**CI at ED90**	**CisPtDm (IC50)**	**Changes of IC_{50} CisPt in mixes *vs.* IC_{50} CisPt alone (fold)**	**p-value**	**Res Dm (IC50)**	**m**	**r**
CisPt (alone)				13.51±1.13 (10.70-16.32)				2.46±0.22 (1.91-3.02)	0.99±0.0 (-)
Res (alone)							74.32±4.27 (63.72-84.92)	2.47±0.13 (2.15-2.80)	0.97±0.01 (0.93-1.00)
Mix 0/0h (a)	1.4±0.15 (1.02-1.77)	1.22±0.12 (0.92-1.52)	1.07±0.10 (0.82-1.31)	9.40±0.42 (8.36-10.44)	1.44±0.18 (1.00-1.89)	NS	51.68±2.30	3.56±0.29 (2.84-4.28)	0.99±0 (0.98-1.00)

							(45.96-57.41)		
Mix 2/0h (b)	2.04±0.12 (1.75-2.34)	1.74±0.14 (1.39-2.08)	1.48±0.15 (1.09-1.86)	13.76±0.43 (12.7-14.83)	0.98±0.07 (0.81-1.15)	NS	75.71±2.36 (69.85-81.56)	3.92±0.27 (3.26-4.58)	0.99±0.0 (0.98-1.0)
Mix 0/2h (c)	1.95±0.15 (1.58-2.23)	1.70±0.15 (1.34-2.06)	1.49±0.14 (1.13-1.84)	13.10±0.26 (12.4-13.75)	1.03±0.09 (0.80-1.26)	NS	72.04±1.45 (68.43-75.65)	3.55±0.2 (3.05-4.06)	0.99±0.0 (0.99-1.0)
Mix median effect	1.8±0.35 (0.94-2.66)	1.55±0.29 (0.83-2.27)	1.35±0.24 (0.83-2.27)	12.09±2.35 (0.75-1.94)	1.15±0.25 (0.52-1.78)	NS	66.48±12.95 (34.32-98.63)	3.68±0.21 (3.15-4.20)	0.99±0.0 (-)
		**TOV-112D**					**Constant ratio (IC_{50}'s ratio) CisPt:Res = 1:32**		
	**CI at ED50**	**CI at ED75**	**CI at ED90**	**CisPt Dm (IC50)**	**Changes of IC_{50} CisPt in mixes *vs.* IC_{50} CisPt alone (fold)**	**p-value**	**Res Dm (IC50)**	**m**	**r**
CisPt				3.25±0.16 (2.86-3.65)				4.12±0.55 (2.76-5.48)	0.99±0.0 (-)
Res							102.92±2.10 (97.72-108.13)	2.42±0.49 (1.2-3.65)	0.97±0.01 (0.94-1.0)
Mix 0/0h (a)	1.34±0.01 (1.3-1.37)	1.28±0.06 (1.14-1.43)	1.25±0.12 (0.95-1.55)	2.16±0.01 (2.12-2.20)	1.51±0.06 (1.35-1.66)	<0.001	67.92±0.47 (72.16-70.27)	3.42±0.06 (3.26-3.58)	0.96±0.0 (0.95-0.97)

Mix 2/0h (b)	1.38±0.01 (1.36-1.40)	1.37±0.10 (1.12-1.62)	1.38±0.19 (0.92-1.84)	2.23±0.05 (2.1-2.36)	1.46±0.04 (1.36-1.55)	<0.001	71.46±1.68 (67.27-75.64)	3.11±0.15 (2.73-3.48)	0.95±0.03 (0.88-1.01)
Mix 0/2h (c)	1.7±0.07 (1.54-1.87)	1.74±0.04 (1.64-1.83)	1.80±0.14 (1.44-2.15)	2.47±0.07 (2.57-2.90)	1.31±0.08 (1.10-1.51)	0.0012	87.66±2.13 (82.36-92.96)	2.76±0.19 (2.30-3.23)	0.95±0.0 (0.94-0.96)
Mix median effect	1.47±0.2 (0.98-1.97)	1.46±0.24 (0.87-2.06)	1.47±0.29 (0.76-2.19)	2.29±0.16 (1.88-2.69)	1.43±0.10 (1.17-1.69)	<0.001	75.68±10.52 (49.53-101.83)	3.10±0.33 (2.28-3.92)	0.95±0.01 (0.94-0.97)

Abbreviations: Data expressed in mean ± SD and in (95%CI= confidence interval); CisPt = cisplatin; Res = resveratrol; Mix = CisPt + Res; CI = combination index at ED50 (50% effect doses), ED75 (75% effect doses) and ED90 (90% effect doses); Dm = the median effect dose of drug; IC_{50} = a dose that produces the half-maximum response Dm=IC_{50} [μM] ; m = an exponent of sigmoidicity (shape) of the dose-effect curve; r = the linear correlation coefficient of the median-effect plot; mix 0/0 h=CisPt and Res added at the same time; mix 2/0 h= Res given 2 hours before CisPt; mix 0/2 h= Res given 2 hours after CisPt. P-value of difference statistically significant or not at p<0.05 has been determined by Student's *t*-test and Mann-Whitney U test; NS = statistically non-significant.

References

95. Stoer, J., Bulirsch,R. Topics in integration In *Introduction to Numerical Analysis*; Springer-Verlag New York: 2002; pp. 145-189.
96. Süli, E., Mayers, D. Numerical integration In *An introduction to numerical analysis* Cambridge University Press: 2003; pp. 220-223.
97. Burden, R.L., Faires, J.D. Numerical differentiation and integration In *Numerical analysis* Boston, MA : Brooks/Cole, Cengage Learning: 2011; pp. 173-258.
98. Zarowski, C.J. Numerical integration and differentiation In *An introduction to numerical analysis for electrical and computer engineers* John Wiley & Sons: 2004; pp. 369-414.
99. Provencher, D.M.; Lounis, H.; Champoux, L.; Tetrault, M.; Manderson, E.N.; Wang, J.C.; Eydoux, P.; Savoie, R.; Tonin, P.N.; Mes-Masson, A.M. Characterization of four novel epithelial ovarian cancer cell lines. *In Vitro Cell Dev Biol Anim* **2000**, *36*, 357-361, doi:10.1290/1071-2690(2000)036<0357:COFNEO>2.0.CO;2.
100. Taniguchi, T.; Tischkowitz, M.; Ameziane, N.; Hodgson, S.V.; Mathew, C.G.; Joenje, H.; Mok, S.C.; D'Andrea, A.D. Disruption of the Fanconi anemia-BRCA pathway in cisplatin-sensitive ovarian tumors. *Nat Med* **2003**, *9*, 568-574, doi:10.1038/nm852.
101. Domcke, S.; Sinha, R.; Levine, D.A.; Sander, C.; Schultz, N. Evaluating cell lines as tumour models by comparison of genomic profiles. *Nat Commun* **2013**, *4*, 2126, doi:10.1038/ncomms3126.
102. Treeck, O.; Schuler, S.; Haring, J.; Skrzypczak, M.; Lattrich, C.; Ortmann, O. icb-1 Gene counteracts growth of ovarian cancer cell lines. *Cancer Lett* **2013**, *335*, 441-446, doi:10.1016/j.canlet.2013.02.049.
103. Stordal, B.; Timms, K.; Farrelly, A.; Gallagher, D.; Busschots, S.; Renaud, M.; Thery, J.; Williams, D.; Potter, J.; Tran, T.; et al. BRCA1/2 mutation analysis in 41 ovarian cell lines reveals only one functionally deleterious BRCA1 mutation. *Mol Oncol* **2013**, *7*, 567-579, doi:10.1016/j.molonc.2012.12.007.
104. Solar, P.; Sytkowski, A.J. Differentially expressed genes associated with cisplatin resistance in human ovarian adenocarcinoma cell line A2780. *Cancer Lett* **2011**, *309*, 11-18, doi:10.1016/j.canlet.2011.05.008.
105. Cimoli, G.; Malacarne, D.; Ponassi, R.; Valenti, M.; Alberti, S.; Parodi, S. Meta-analysis of the role of p53 status in isogenic systems tested for sensitivity to cytotoxic antineoplastic drugs. *Biochim Biophys Acta* **2004**, *1705*, 103-120, doi:10.1016/j.bbcan.2004.10.001.
